# Triple‐Mode Ferroelectric Thin‐Film Transistor for Hybrid Electrical–Optical Reservoir Computing

**DOI:** 10.1002/advs.75471

**Published:** 2026-05-11

**Authors:** Hyeonho Lee, Seungjun Lee, Tae‐Hyeon Kim, Yonghee Jeong, Gwangmin An, Minsu Ko, Junsu Yu, Heung Soo Kim, Yang Chai, Sungjun Kim

**Affiliations:** ^1^ Division of Electronics and Electrical Engineering Dongguk University Seoul Republic of Korea; ^2^ Department of Applied Physics The Hong Kong Polytechnic University Kowloon Hong Kong China; ^3^ Department of Semiconductor Engineering Seoul National University of Science and Technology Seoul Republic of Korea; ^4^ School of Electrical and Computer Engineering Georgia Institute of Technology Atlanta Georgia USA; ^5^ Department of Mechanical Robotics and Energy Engineering Dongguk University Seoul Republic of Korea

**Keywords:** ferroelectric thin‐film transistor, field effect, hafnium zirconium oxide, indium gallium zinc oxide, neuromorphic device, reservoir computing, triple mode memory

## Abstract

This study proposes a fully hardware‐based reservoir computing (RC) system, which utilizes a single ferroelectric thin‐film transistor (FeTFT) based on a Si_3_N_4_/Hf_0.5_Zr_0.5_O_2_ (HZO)/indium gallium zinc oxide (IGZO) tri‐layer stack. The proposed FeTFT operates in three independent memory modes according to two different input signals, electrical stimulation and optical stimulation: electric long‐term (LT), electric short‐term (ST), and optical ST. Through this, non‐volatile memory characteristics and volatile memory characteristics were successfully integrated within a single device. The non‐volatile electric LT mode is based on the ferroelectric polarization switching mechanism of the HZO layer and was utilized as the readout layer for the RC, demonstrating excellent endurance and retention characteristics. The volatile electric ST mode is based on the charge trapping mechanism of the Si_3_N_4_ trap layer, while the optical ST mode is based on the optical ionization mechanism of the IGZO channel. These two ST modes perform the function of a 4‐bit reservoir layer with short‐term characteristics such as paired‐pulse facilitation. Finally, the fully hardware‐based RC system, which organically integrates these three modes and applies the hybrid mapping model, achieved a high recognition accuracy of 92.43% in the Modified National Institute of Standards and Technology dataset recognition task.

## Graphical abstract

A triple‐mode ferroelectric thin‐film transistor is developed by integrating Si_3_N_4_/HZO/IGZO layers to realize three independent memory modes: electric long‐term, electric short‐term, and optical short‐term. This single‐device architecture functions as both a reservoir and readout layer, achieving 92.43% MNIST accuracy. It offers a fully hardware‐based, energy‐efficient neuromorphic system with 11.6 pW power consumption and enhanced dynamic diversity.

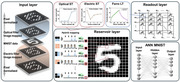

## 1 Introduction

In today's data‐intensive world, advances in artificial intelligence (AI) require computing architecture to meet the escalating computational needs. However, conventional computation systems, characterized by the distinct separation between memory and processing units, are challenged by frequent data transfer delays, resulting in substantial power consumption and reduced processing speed [1, 2]. Among various approaches, neuromorphic computing inspired by human neural systems has emerged as a core driver for next‐generation AI applications [3, 4, 5]. In addition, neural network performs multiple tasks simultaneously with parallel processing and low power consumption. As these network models have matured, their systems have spread dramatically into a variety of fields, including autonomous driving [6], biological science [7, 8], and robotics [7, 8, 9, 10]. However, complex and high‐volume information requires massive network interactions between neurons and synapses, leading to significant energy inefficiency [11, 12]. Reservoir computing (RC) stands out as a promising model well‐suited for nonlinear and time series tasks such as speech recognition [13], weather forecasting [14, 15], and pattern recognition [16, 17]. RC consists of a three‐layer architecture: an input layer which pre‐processes the information, a reservoir that projects the signal into a high‐dimensional space, and a readout layer that extracts relevant features from reservoir states. Unlike other recurrent structures, RC simplifies the learning process by fixing the inherent parameters of the reservoir, thereby reducing computational cost [18, 19]. The short‐term memory (STM) characteristics of the reservoir play a crucial role in capturing the temporal correlations within sequential data, enabling effective pattern prediction and dynamic behavior mapping. In recent years, several approaches have been explored to implement physical reservoirs using memristors [20, 21], ferroelectric thin‐film transistor (FeTFT) [22], and memtransistors [23, 24]. These approaches provide potential advantages regarding scalability and low operating voltage. However, devices capable of simultaneously exhibiting STM and long‐term memory (LTM) properties, suitable for each reservoir and readout layer, remain scarce. Although some studies have proposed separate devices to implement RC systems, these approaches often involve a difficulty of fabrication and fine‐tuning process [25, 26]. To address these challenges, Hafnium zirconium oxide (HZO)‐based FeTFT has gained attention as a candidate for implementing RC systems [27, 28, 29].

**FIGURE 1:**
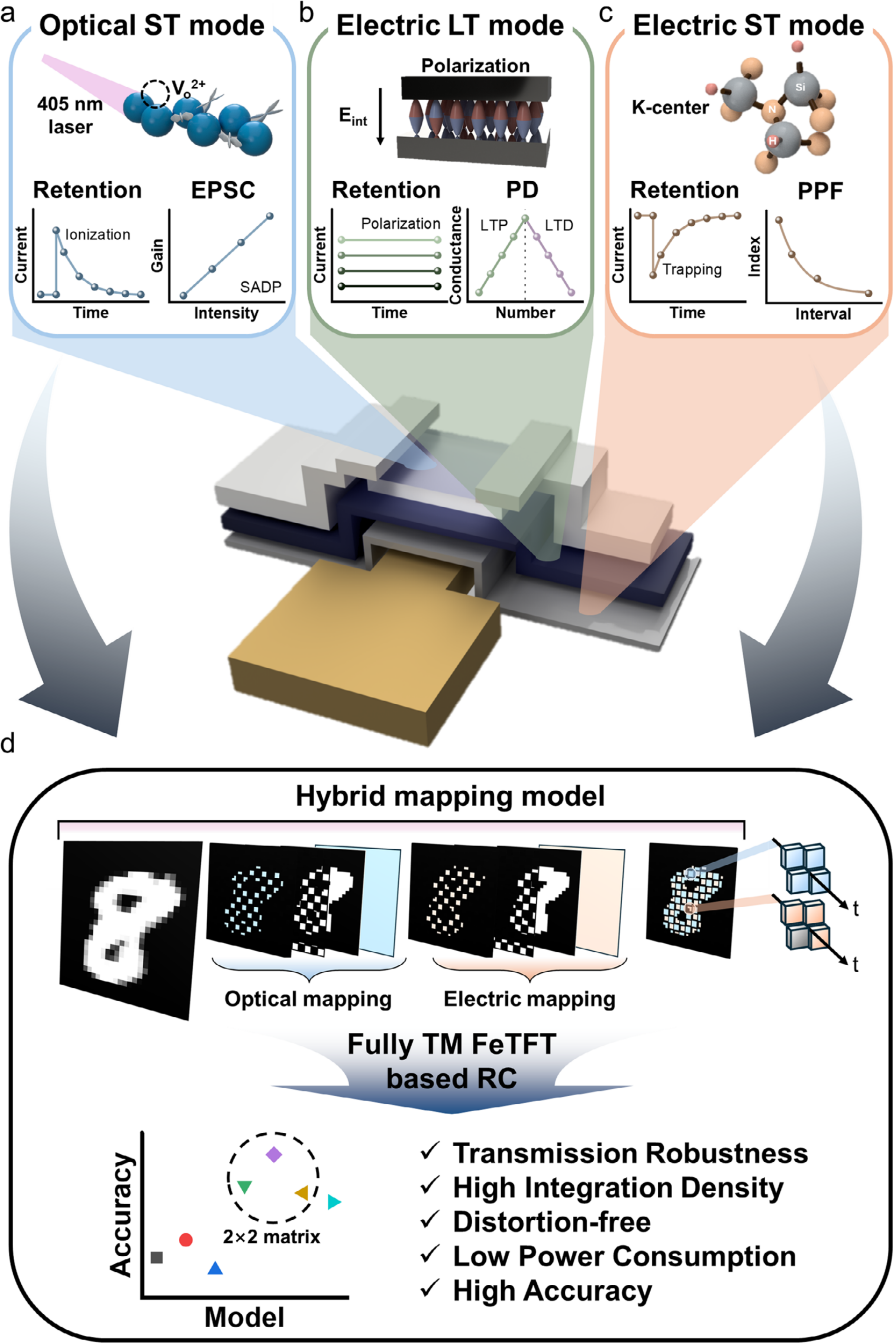
Layer‐specific mechanisms and memory characteristics of the TM FeTFT. (a) Optical ST mode. (b) Electric LT mode. (c) Electric ST mode. (d) Schematic of the Hybrid mapping model utilizing two STM modes implemented in the fully TM FeTFT‐based RC system, and a conceptual illustration emphasizing its advantages.

In this study, we fabricated an indium gallium zinc oxide (IGZO) channel FeTFT incorporating a Si_3_N_4_/HZO gate insulator structure as a multifunctional device. As illustrated in Figure 1a, the IGZO channel, with its wide bandgap (3.4 eV), high mobility, and excellent light‐responsive properties, enables fading memory behavior by modulating optical pulses [30, 31, 32]. Figure 1b presents the HZO film exhibiting polarization dynamics and polarization charge coupling, which enables control of multilevel nonvolatile states [33]. While recent studies utilizing simple two‐terminal memristors or organic synaptic transistors have reported RC systems achieving over 90% accuracy, two‐terminal architectures inherently suffer from severe sneak‐path currents and destructive reads during high‐density array integration. Furthermore, there are limitations to a true low‐power, fully hardware‐based implementation, as the readout layers rely on external software simulations or exhibit relatively high‐power consumption during reservoir operation [16, 34]. In contrast, three‐terminal ferroelectric thin‐film transistors (FeTFTs) provide structurally separated signal paths, ensuring reliable non‐destructive weight updates. While the individual physical mechanisms, such as ferroelectricity in HZO, charge trapping in Si_3_N_4_, and photoionization in IGZO, are widely known, and several previous studies have attempted to integrate them into two‐mode systems by combining HZO polarization with tunneling‐based charge trapping or optical responses, successfully integrating all three independent mechanisms within a single device has remained a significant challenge [35, 36]. However, implementing a fully hardware‐based RC system using a single three‐terminal device without any stack variations between the reservoir and readout layers, operating at a picowatt (pW)‐level ultralow power such as 11.6 pW, and completely eliminating the reliance on external software, while achieving over 90% accuracy in 4‐bit based MNIST recognition tasks, has not yet been reported. To overcome these limitations and enhance accuracy, we designed a hybrid reservoir by introducing a Si_3_N_4_ layer alongside the conventional ferroelectric layer to induce additional STM characteristics via charge‐trapping mechanisms (Figure 1c) [37, 38, 39]. This approach goes beyond merely bi‐directionally expanding the memory window (MW) for improved LTM characteristics; it strategically combines the fast dynamics based on charge trapping with the slow dynamics induced by the optical ionization of the IGZO channel. The integration of such heterogeneous time constants maximizes the dynamical diversity of the reservoir, enabling more efficient mapping of complex time‐series data into a high‐dimensional feature space, effectively realizing all these features within a single gate stack device. The time‐dependent characteristics arising from three distinct mechanisms of the triple‐mode (TM) FeTFT were verified through electrical and optical measurements, confirming ferroelectric properties, *I*_ds_–*V*_gs_ characteristics, and potentiation/depression (PD) behavior over 10 cycles. STM behavior was further evaluated using paired‐pulse facilitation (PPF), excitatory postsynaptic current (EPSC), and retention tests. We demonstrated the separability of 16 states implemented in a 4‐bit reservoir state RC system. To maximize accuracy, we propose a hybrid mapping model with increased complexity by combining mapping methods with two STM pulses generated by electrical and optical stimuli and a 2 × 2 matrix. Furthermore, to achieve high pattern recognition accuracy, we propose a hybrid mapping model that enhances the effective rank (ER) by combining two STM pulses generated by electrical and optical stimuli with a 2 × 2 matrix. Consequently, we implemented a fully TM FeTFT‐based RC system combined with the hybrid mapping model and incremental PD. The accuracy was evaluated via image classification using the MNIST dataset with an ANN. Beyond high accuracy, this RC system offers advantages in transmission robustness attributed to mutual complementary interactions, high integration density unconstrained by optical equipment size, distortion‐free operation resulting from the 2 × 2 matrix, and low power consumption of 11.6 pW utilizing light, offering high potential for physical‐RC systems.

## 2 Result and Discussion

### 2.1 Non‐Volatile Memory Properties of FeTFT Utilizing a Ferroelectric HZO Layer

**FIGURE 2:**
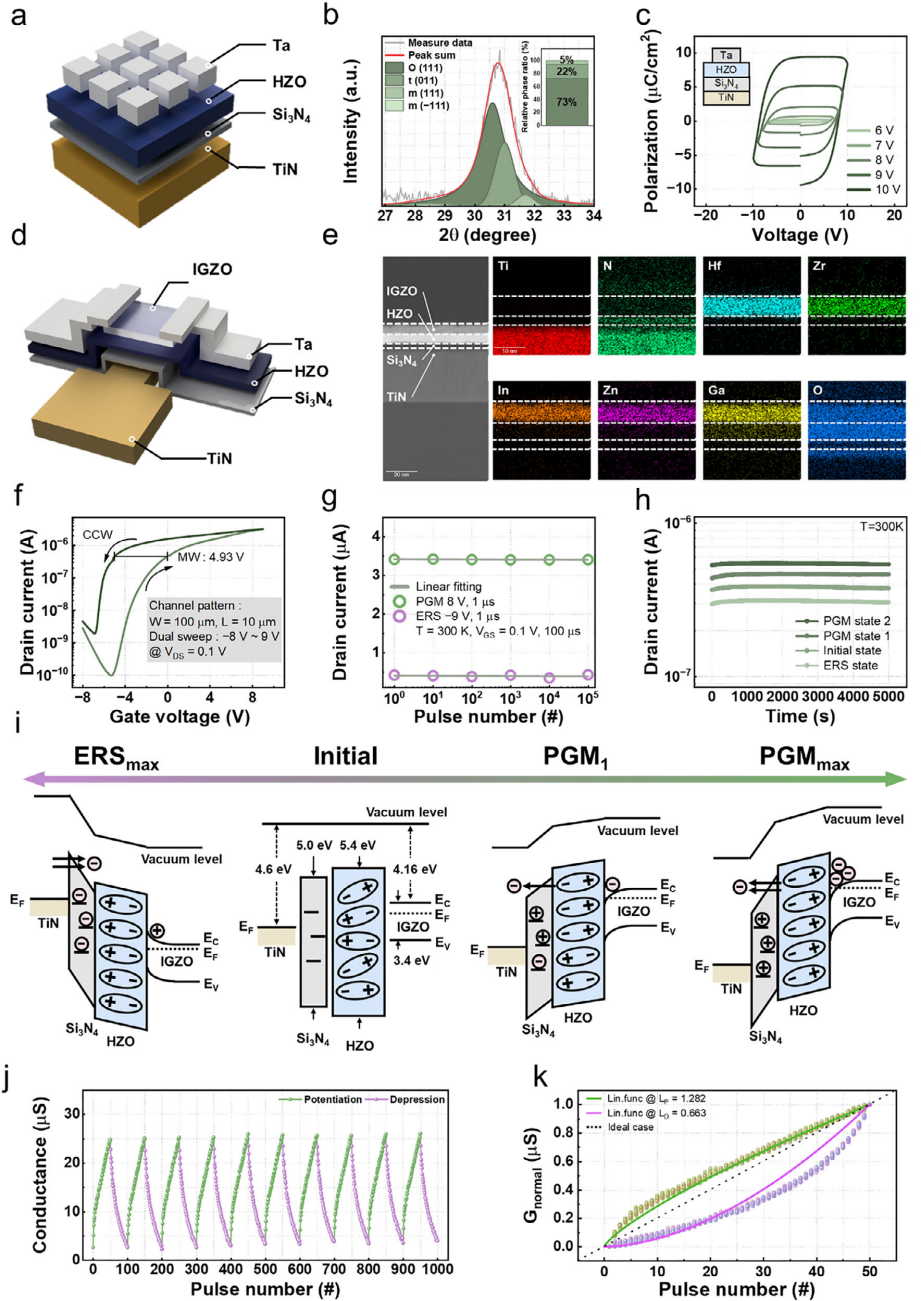
(a) Schematic of the TiN/Si_3_N_4_/HZO/Ta FeCAP device structure. (b) GIXRD analysis of the FeCAP. (c) *P*–*V* characteristics of the FeCAP. (d) Schematic of the FeTFT device structure. (e) TEM and EDS analysis of the FeTFT. (f) Dual‐sweep *I*_ds_–*V*_gs_ transfer curve of the FeTFT. (g) Endurance characteristics over 10^5^ cycles for PGM and ERS states. (h) Retention characteristics of four states measured over 5000 s. (i) Schematic diagram of the gate stack illustrating the operating mechanism in the HZO ferroelectric layer under an applied electric bias. (j) LTP and LTD characteristics obtained via a 10 cycles incremental pulse scheme. (k) Normalized and overlapped 10 cycles PD for the incremental pulse scheme, shown with the corresponding linearity function.

In the FeTFT gate stack, the HZO layer is the most fundamental layer responsible for the polarization characteristics. For a precise analysis of the HZO layer, a ferroelectric capacitor (FeCAP) with a stack identical to the gate stack was fabricated prior to the three‐terminal device process. Figure 2a shows a general schematic of this Metal‐Insulator‐Ferroelectric‐Metal (MIFM) FeCAP, illustrating a TiN/Si_3_N_4_/HZO/Ta stack. All capacitor devices fabricated in this study share a TiN bottom electrode and have a top electrode patterned to a size of 100 µm × 100 µm, as confirmed by the critical dimension scanning electron microscopy (CD‐SEM) image in Figure S1a. It is well‐known that for HZO‐based ferroelectrics, the polarization characteristics are significantly improved when tensile stress is applied by performing post‐metallization annealing (PMA) after the capping layer deposition [40]. Accordingly, tungsten, which has a lower coefficient of thermal expansion than HZO and can thus induce tensile stress upon deposition [41, 42], was used as the capping layer, followed by annealing at 500°C for 30 s. To identify the dominant crystal structure of the HZO layer stressed by PMA, grazing incidence X‐ray diffraction (GIXRD) analysis was performed targeting the HZO layer of this W‐capped FeCAP, as shown in Figure 2b. The measurement was conducted in the 2*θ* angular range of 27° to 34°. The analyzed data represents a convoluted value consisting of the m(−111), o(111), t(011), and m(111)‐phase peaks, which are present at approximately 28.5°, 30.6°, 31.0°, and 31.7°. For the quantitative analysis of the orthorhombic phase, which is responsible for ferroelectricity, the measured data was deconvoluted into individual peaks using Gaussian fitting. Based on the extracted area of each peak, an analysis of the overall phase ratio revealed that the orthorhombic phase accounted for more than half (73%), indicating the suitability of the HZO layer for use as a ferroelectric material. To evaluate the dominant polarization characteristics and determine the remnant polarization (*P*_r_) value of the HZO, *P*–*V* curves were derived in Figure 2c based on the current response obtained from the positive‐up‐negative‐down (PUND) measurement method. The specific PUND pulse scheme applied to the top electrode and the corresponding current response to the maximum PUND voltage are shown in Figure S2a,b. To exclude the influence of the initial state, [N] and [D] pulses with the same amplitude and duration were applied prior to the PUND pulse scheme to align the dipoles. Upon the application of the first [P] and [N] pulses, a high current peak is observed, which consists of both the switching‐current component due to rapid dipole switching and the non‐switching current component. This peak is higher than that of the [U] and [D] pluses, which contain only the non‐switching current. The responsive current shows a proportional relationship with the maximum PUND voltage, implying that the number of aligning dipoles increases as the voltage magnitude increases. To secure a wide MW in the three‐terminal FeTFT, it is essential to obtain a sufficient coercive voltage (*V*_c_), the voltage required to reverse polarization. The Si_3_N_4_ layer, which has a lower dielectric constant than HZO, effectively reduces the voltage drop across the HZO layer itself. This signifies an extension of the voltage range that can be matched to the polarization value. Ultimately, a 2P_r_ value of 19.44 µC cm^−2^ and a *V*_c_ of 8 V were obtained at under a ±10 V voltage sweep.

Based on the conditions tested with the FeCAP, an MIFS FeTFT with a Ta/Si_3_N_4_/HZO/IGZO/Ta stack was fabricated, and an approximate schematic of the FeTFT stack is shown in Figure 2d, with the top view of the fabricated device confirmed by the CD‐SEM image in Figure S1b. Figure 2e depicts the cross‐sectional transmission electron microscopy (TEM) image and corresponding energy‐dispersive spectroscopy (EDS) analysis results. Through the TEM image, distinct boundaries separating each layer are observed, and the identified thicknesses are consistent with the gate stack's design thicknesses (Si_3_N_4_ 2 nm/HZO 7 nm/IGZO 10 nm). The constituent materials for each layer were confirmed via EDS. The nitride and oxide components were confirmed to be localized within their intended layers. The Hf and Zr elements of the HZO used as the ferroelectric are distributed together within the 7 nm thick layer, and the In, Zn, and Ga constituting the IGZO channel are also observed together in the 10 nm layer. Figure 2f shows the current response of the 3‐terminal FeTFT according to the gate voltage range. The device's channel pattern is width (*W*)/length (*L*) = 100 µm/10 µm, and all electrical measurements were conducted at *V*_ds_ of 0.1 V. From the forward sweep, an on/off ratio of ≈ 10^4^ was secured, along with an ultra‐low off‐current of 100 pA and a maximum on‐current of ≈ 30 µA. In the backward sweep, the alignment of internal dipoles due to the applied external bias attracts electrons within the IGZO toward the channel, increasing the conductance. This results in a counterclockwise (CCW) *I*−*V* hysteresis, and a large *M*_W_ of 4.93 V was observed as a result of the voltage sweep up to 9 V. Figure 2g presents the endurance evaluation results for the states separated by polarization. The selected program (PGM) at *V*_gs_ of 8 V for 1 µs and erase (ERS) at *V*_gs_ of −9 V for 1 µs are conditions experimentally chosen to enable consistent state transitions. After PGM and ERS, measurements were taken by applying a 0.1 V, 100 µs gate read pulse to the TiN gate. After plotting representative values at decade intervals and performing linear fitting, the slopes for both states were found to be below 10^−9^. During 10^5^ repetitive cycles, a maximum state shift of 0.38% was observed for the initial PGM state, and a 6.22% state shift was observed for the ERS state, demonstrating excellent endurance and reliability. To test the retention characteristics at 5000 s by additionally adjusting states at uniform intervals from the initial aligned state, 5000 s retention measurements were conducted at 300 K for four selected states, as depicted in Figure 2h. The read condition involved applying a fixed voltage bias of *V*_gs_ = 0 V and *V*_ds_ = 0.1 V, with reads performed at 3 s intervals. The measurement results confirmed that all four states showed no interference effects up to 5000 s and were clearly separated. Figure 2i explains the multi‐state generation mechanism, arising from screening and polarization characteristics, using a schematic band diagram of the gate stack. In the Initial state, the dipoles within the HZO are unaligned and point in random directions. The moment a positive voltage is applied, the dipoles rotate in proportion to the applied voltage, and simultaneously, electrons trapped in low‐energy states in the Si_3_N_4_ tunnel and move to the gate TiN. The positive charge in the form of empty electron states at the trap states and the Si_3_N_4_/HZO interface forms a pinning with the dipoles inside the HZO. Finally, upon reaching the max state, all trap states are emptied, and all dipoles point toward the channel, bending the IGZO band downward at the interface and attracting more charge to the channel. Subsequently, when attempting to reduce the state via an ERS pulse, the charges pinned at the interface and the Si_3_N_4_ charge screen the bias applied to the HZO from the external bias. This screening effect, in addition to the voltage division from the dielectric constant ratio, causes more energy to be consumed per gate volt to rotate the dipoles than initially, which is a direct cause of the wide MW [43, 44]. An ERS pulse above the threshold conversely supplies electrons from the TiN via tunneling, increasing the amount of negative charge stored at the top of the HZO, which bends the IGZO band upward and decreases the channel conductance.

To implement hardware‐based RC, devices functioning as neurons and synapses with distinct characteristics are required. Synapses serve as mediators that transmit signals from presynaptic neurons to postsynaptic neurons. Just as biological synapses flexibly adjust their connection strength in response to various stimulus, the device implemented in the electric long term (LT) mode must be capable of updating its conductance linearly and symmetrically in response to electrical stimulation. In Figure S3e,a, the drain current is plotted as conductance after applying 50 potentiation pulses and 50 depression pulses for 10 cycles. The identical pulse scheme applied to the gate is shown in Figure S3b, consisting of 50 potentiation pulses at 7 V for 5 ms and 50 depression pulses at −9 V for 1 ms applied to the TiN gate. 500 ms after each LT pulse application, a *V*_ds_ of 0.1 V and a *V*_gs_ of 1 V were applied for the read operation. When evaluating the long‐term potentiation (LTP) and long‐term depression (LTD) characteristics using these uniform pulses, the conductance change sharply decreases after the initial pulse, as the majority of dipoles have already reacted. Particularly in the potentiation phase, while the first 10 states exhibit a 20 µS shift range, the states near the 40th pulse are separated by only 10 µS, clearly demonstrating non‐linear characteristics. This bunched conductance state phenomenon can cause errors in weight updates during neural network training or slow down the learning speed required to find appropriate weights. Therefore, to strictly examine the linearity by obtaining a linearity factor, the following equation was applied [45, 46, 47].

(1) $$G\left(P\right)={\left(\left(\alpha {\left(P\right)}^{L}-\beta {\left(P\right)}^{L}\right)\times w+\beta {\left(P\right)}^{L}\right)}^{\frac{1}{L}}$$

(2) $$\alpha {\left(P\right)}^{L}=B\left(1-e^{-\frac{P}{A}}\right)+G_{\min}$$

(3) $$\beta {\left(P\right)}^{L}=-B\left(1-e^{-\frac{P-P_{\max}}{A}}\right)+G_{\max}$$

(4) $$B=\frac{G_{max}-G_{\min}}{1-e^{-\frac{P_{\max}}{A}}}$$

α(*P*) and β(*P*) represent the conductance during LTP and LTD, respectively, with the number of pulses *P* as the independent variable. *w* is an internal variable that moves between 0 and 1 with each long‐term pulse application. *G*_max_ and *G*_min_ are the maximum and minimum conductance values across all *P*, and *B* normalizes the conductance within this allowed range. *P*_max_ is the maximum *P* at which saturation occurs, and the parameter *A* controls the degree of saturation caused by the exponential movement. *L* is the linearity factor, which is an approximated value that minimizes the error between the measured data over all pulses and the linearity function. Figure S3f shows the identical 10‐cycle PD, consisting of 50 points each based on *P_max_*, which was approximated by applying the function, yielding *L*_P_ = 4.047 and *L*_D_ = 0.542. Unlike a linearity factor obtained simply from the magnitude of the error relative to the ideal value, the linearity factor obtained by approximating this function includes information such as the graph's shape, or concavity, saturation point, and error magnitude. Linearity factor values between 0 and 1 have a concave‐up shape, and as the linearity value approaches 1, the saturation point increases. A linearity factor of 1 signifies a perfectly linear state, which is the ideal case. For linearity values greater than 1, the saturation point decreases as the value increases, resulting in a concave‐down shape. To improve linearity, the incremental pulse scheme shown in Figure S3c,d was used instead of the identical pulse scheme applied to the TiN gate. This method, where the amplitude starts 2 V lower and gradually increases by 40 mV, effectively suppresses the dipole response to the initial pulses, and the 40 mV incremental pulse provides additional margin compared to the existing conductance increase. In terms of stability, as presented in Figure S3a, the identical method is dominated by the initial polarization response. Errors in this region accumulate as pulses continue, causing state shift and interference with other states. In contrast, the incremental method, despite having lower peak conductance, prevents the deviation in each state from carrying over to other states due to its good linearity factor. Using this incremental approach, the conductance response over 10 cycles was recorded, as shown in Figure 2j. For a more detailed comparison, the linearity function was used in the same manner. In Figure 2k, the incremental 10‐cycle PD was plotted, again with 50 points each based on *P*_max_, and approximated with *L*_P_ = 1.282 and *L*_D_ = 0.683. Consequently, both *L*_P_ and *L*_D_ were significantly improved, moving much closer to the ideal value of 1 than before. This high linearity was achieved not only by the incremental amplitude but also by maintaining a 500 ms read delay after each pulse. This delay, corresponding to approximately three times the slow relaxation time constant, effectively minimizes the discrepancy in transient decay induced by the gradually varying pulse voltage, thereby isolating the non‐volatile LT characteristics. The partial polarization characteristics, arising from high electrical stimulation in the Si_3_N_4_ and HZO layers, result in multi‐level states that are retained without additional bias. Particularly when using the incremental pulse scheme, the device exhibits linear states, allowing for the adjustment of appropriate weights in fewer attempts, confirming its suitability as a readout layer. To further evaluate the practical viability of the incremental pulse scheme, we systematically investigated its system‐level trade‐offs, specifically regarding energy consumption and programming time. The energy dissipated during programming was calculated by multiplying the measured gate current, the applied gate voltage, and the pulse duration. Because the incremental method initiates programming at a lower voltage amplitude and gradually steps up, the overall energy dissipated across the sequence is fundamentally reduced. Averaging over 10 cycles, the total energy consumption for the identical pulse scheme was 18.5 nJ for potentiation and 4.72 nJ for depression, whereas the incremental scheme recorded lower values of 15.9 and 4.22 nJ, respectively. This demonstrates a 14.05% reduction in potentiation energy and a 10.59% reduction in depression energy (Figure S4a). We further investigated the programming speed. As the pulse width and intervals are identical in both schemes, the programming time is strictly dictated by the number of pulses required to reach a target state. We measured the time to reach specific normalized conductance levels from 0 to 1 starting from a 0.5 baseline. In the range from 0 to 0.5, where only erase pulses are applied, the programming times between the two schemes are highly comparable. However, reaching higher target states above 0.5 requires significantly more time with the incremental scheme due to the gradual voltage steps. When summing the time required for all normalized target states, the identical scheme takes 482.9 ms, whereas the incremental scheme takes 620.6 ms, making it approximately 28.5% slower overall (Figure S4b). Nevertheless, in the context of a hardware‐based reservoir computing readout layer, the precise tuning of synaptic weights is paramount. The drastic improvement in conductance linearity and the simultaneous reduction in total energy consumption render this moderate increase in programming time a highly justifiable and advantageous trade‐off for the system.

### 2.2 Volatile Memory Properties of FeTFT Utilizing a Si_3_N_4_ Trap Layer

**FIGURE 3:**
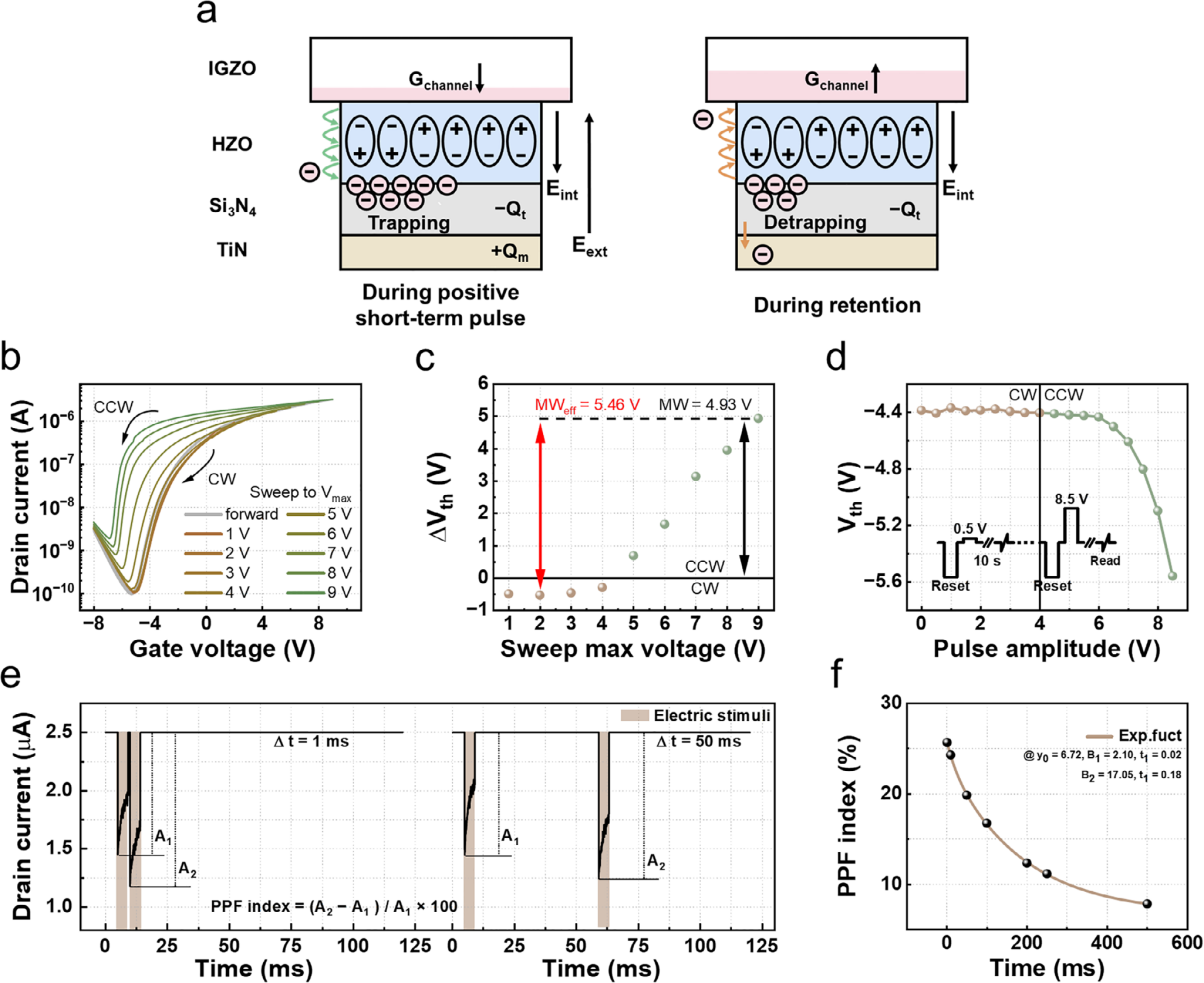
(a) Schematic diagram of the gate stack illustrating the electron trapping/detrapping mechanism in the Si_3_N_4_ trap layer after pulse application. (b) Dual‐sweep *I*_ds_–*V*_gs_ transfer curves as a function of varying maximum voltage (*V*_max_) swept from −8 V in 1 V increments. (c) Corresponding *V*_th_ shift vs *V*_max_. (d) *V*_th_ after a 10 s delay versus increasing pulse amplitude in 0.5 V increments. (e) Current response to paired electrical pulses applied at intervals of 1 and 50 ms. (f) Dependence of the PPF index on the pulse interval time.

Figure 3a is a gate stack diagram explaining the short‐term (ST) phenomenon that operates by electron trapping and detrapping in the Si_3_N_4_ trap layer. In an initial state with four dipoles oriented upwards, electrons present in the HZO and IGZO are injected into both the trap layer and the HZO layer via tunneling during the application of a positive, low‐amplitude ST pulse, leading to electron loss in the channel [35, 48]. The trapped charge (*Q*_t_) at the Si_3_N_4_/HZO interface and within the Si_3_N_4_ internal states, possessing a negative sign, flattens the downward‐bent IGZO band and repels channel electrons toward the bulk region, thereby reducing the initial channel conductance below the reference value. Since there is no large bandgap barrier layer with a significant band offset on either side of the trap layer, *Q*_t_ gradually decreases over time as it migrates to the TiN gate or the HZO [49, 50]. As *Q*_t_ gradually diminishes during retention, the decreasing negative charge bends the IGZO band downward again and increases the electrons accumulated in the channel. The conductance during sustained retention is ultimately dependent on the amount of *Q*_t_. A peak is formed immediately after the pulse due to the trapping induced by the small ST pulse, and the state eventually returns to the initial state over time. This explains why the short‐term mechanism appears at low bias. To distinguish the boundary between the CCW and CW sweeps under the same electrical stimulation and to confirm the *V*_th_ shift caused by *Q*_t_, a direct current (DC) dual sweep was measured, as shown in Figure 3b [51]. The voltage condition involved sweeping the TiN gate with incrementally increasing maximum voltages in 1 V steps within the positive operating range of the device, and the read was conducted at *V*_ds_ = 0.1 V. To ensure comparison from the same initial state, the *V*_th_ value was reset by applying an initialization pulse after each measurement. The individual backward responses corresponding to this matched forward sweep are illustrated in Figure S5. Figure 3c plots the *V*_th_ shift (Δ*V*_th_) extracted from the previous current response data. Below 4 V, a CW sweep is confirmed, indicating that the conductance change due to trapping is dominant. The Δ*V*_th_ increasing in the negative direction at 1 V shows that the effect of stored negative charge is stronger than the polarization characteristic from the bias. It reaches a peak at 2 V, and after 4 V, the ferroelectric influence becomes dominant. Consequently, the observed clockwise (CW) hysteresis and the quadratic trend with respect to the sweep maximum voltage suggest that these behaviors are not merely governed by the voltage magnitude, but are fundamentally attributed to the trade‐off relationship between the trapped charge originating from the trap layer and the ferroelectric polarization characteristics.

Figure 3d presents the result of the *V*_th_ response measured for a 1 ms width pulse in an AC environment to confirm the mode change point and ST recovery. The pulse scheme shown in the graph was applied to the gate. After an initial −8 V, 1 ms reset pulse, a reference pulse with a fixed 1 ms width and increasing amplitude in 0.5 V steps was applied. The read was performed by applying 0.1 V to the drain after a 10 s delay. The reset ensured starting from a state with an initial *V*_th_ of −4.4 V. For pulses of 4 V, 1 ms and below, a sufficient time of 10 s was allowed for the channel conductance to decay via electron detrapping. Thus, the saturated value is solely influenced by the polarization characteristics. Below 3 V, *V*_th_ values smaller than −4.4 V were observed, and at 3, 3.5, and 4 V, it was confirmed that the state returned to the initial −4.4 V state. Above 4 V, the influence of HZO is dominant, and a phenomenon of exponential V_th_ shift according to amplitude is observed. To confirm the PPF characteristics arising from the short‐term properties, the current characteristics according to the interval of two ST pulses were investigated, as shown in Figure 3e,f. The ST pulse width and amplitude were applied under the 3 V, 1 ms condition obtained from previous data. The paired‐pulse responses at intervals of 1, 10, 50, 100, 200, 250, and 500 ms can be confirmed in Figure S6a−g. The first electrical stimulation generates the *A*_1_, after which the conductance decays over time. The *A_2_* resulting from the second ST pulse is larger than *A_1_*, due to the incomplete decay following the first stimulus. This behavior is determined by the interval between the two pulses and implements a forgetting process [52, 53]. The PPF index was calculated by the following equation: [36, 54]

(5) $$\mathrm{PPF}\;\mathrm{index}\;\left(\%\right)=\frac{A_{2}-A_{1}}{A_{1}}\times 100$$

*A_1_* and *A_2_* are the relative current fluctuation values obtained from the peak caused by electrical stimulation, relative to the initial state. In the case of a 1 ms interval, the current responding to the second electrical stimulation was confirmed to increase by 25.7% compared to *A_1_*. This is because, as the interval decreases, the pulse is less affected by the relaxation, which decays exponentially. Therefore, the PPF index obtained as a function of the interval can be approximated by the following exponential function model:

(6) $$y=B_{1}\exp\left(\frac{-x}{t_{1}}\right)+B_{2}\exp\left(\frac{-x}{t_{2}}\right)+y_{0}$$

This facilitation phenomenon is composed of two exponential components. *B*_1_ is the initial amplitude of the fast relaxation component, and *t*_1_ is the time constant representing the speed at which this facilitation effect quickly disappears. *B*_2_ is the initial amplitude of the slow relaxation component, and *t*_2 _is the time constant representing the speed at which the facilitation effect gently relaxes. *x* is the variable representing the interval between the two stimuli, and *y*_0_ is a constant. Consequently, the approximated curve was fitted with *y*_0_ = 6.72, *B*_1_ = 2.10, *t*_1_ = 0.02, *B*_2_ = 17.05, and *t*_2 _= 0.18.

### 2.3 Volatile Memory Properties of FeTFT Utilizing IGZO Channel Layer

**FIGURE 4:**
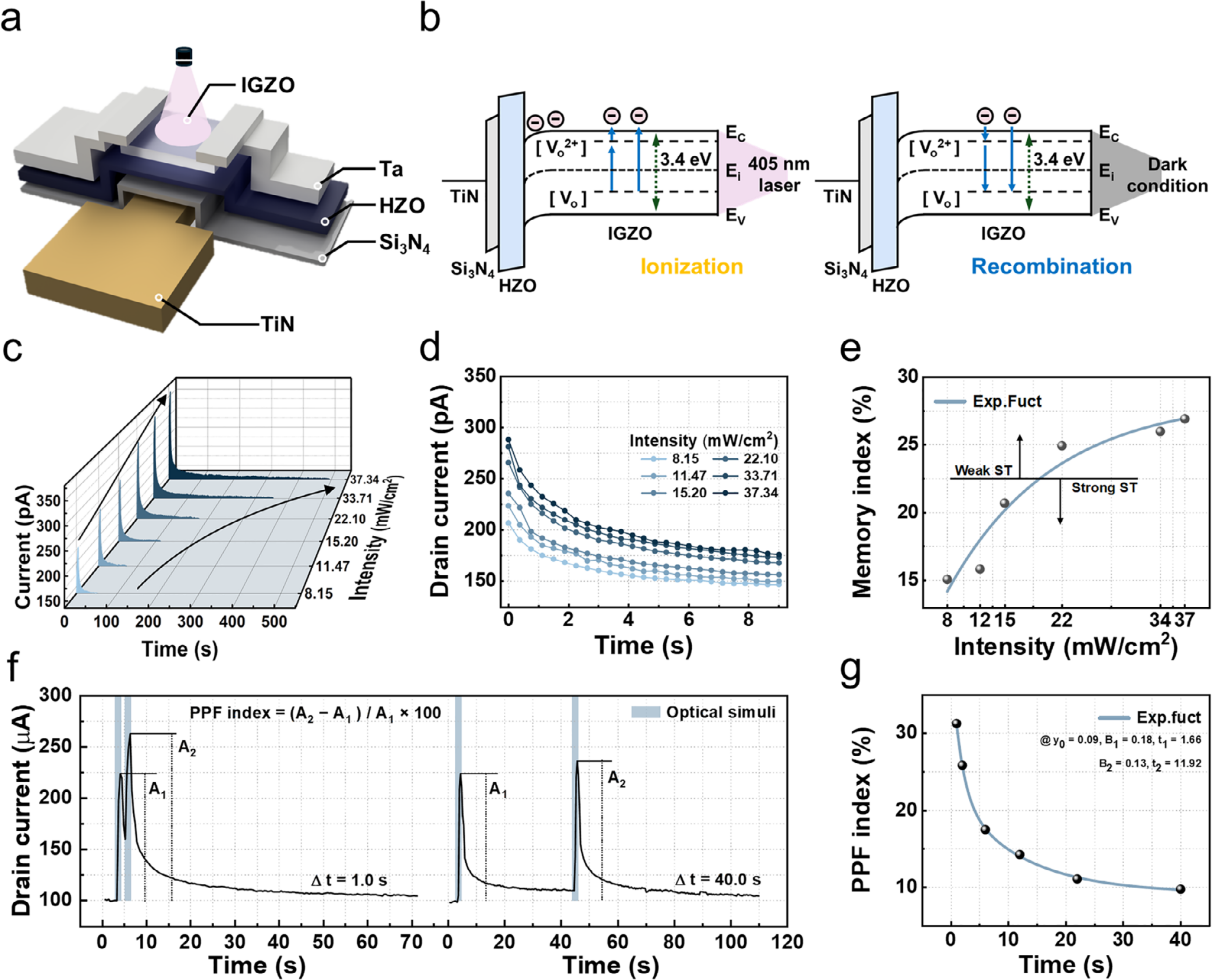
(a) Schematic illustration of the FeTFT device configured for optical operation. (b) Energy band diagrams elucidating the operating mechanism in the IGZO channel, involving ionization under illumination and recombination in the dark. (c) 3D plot displaying the peak current and decay time versus light intensity. (d) Retention characteristics within 10 s after the peak for different light intensities. (e) Memory index properties observed at various current levels, which are dependent on the light's intensity. (f) Temporal current response to paired optical pulses applied at intervals of 1 and 40 s. (g) PPF index plotted against the pulse interval time.

Figure 4a is a schematic showing how the 405 nm laser implements an optical pulse. Due to the bottom‐gate structure, the exposed channel on top can be directly stimulated using optical equipment without considering the absorption coefficient of other layers that would otherwise need to be passed through to reach the IGZO. The scheme for all light measurements was controlled using Thorlabs SC10 software, which controlled the open time corresponding to the optical pulse width down to the millisecond. A dedicated aperture, attached in front of the laser and adjustable with high precision, was used to transmit the light, setting a constant intensity corresponding to the optical pulse amplitude. Figure 4b is a schematic of the overall short‐term mechanism that occurs due to 405 nm optical stimulation. Oxide semiconductors like IGZO possess oxygen vacancy (V_o_) states due to oxygen‐deficient sites during deposition. When *V*_o_ gains energy, it undergoes ionization and acts as a source of electrons via two reactions: V_o_ → V_o_^+^ + e^−^ or V_o_ → V_o_^2+^ + 2e^−^ [55, 56]. In the density of states models for IGZO, V_o_ states have typically been represented as a distribution centered at approximately 2/3 of the total bandgap [57, 58, 59]. Approximately 2.27 eV of external energy is required to break the bond with *V*_o_ in IGZO and reach the conduction band (*E*_c_), thereby affecting the channel conductance. The minimum required wavelength is calculated to be 546 nm by the Planck‐Einstein relation. A 405 nm wavelength laser was selected to elicit reactions from all *V*_o_ states, which are Gaussian‐distributed. While the 405 nm optical stimulation is applied, numerous photoelectrons supplied to *E*_c_ from the ionized *V*_o_ move toward the channel, increasing the conductance and causing a peak‐form to rise in the drain current. After the optical stimulation, under dark conditions where the external energy was blocked, the photoelectrons that had saturated *E*_c_ undergo recombination with the empty V_o_^+^ and V_o_^2+^ states within the IGZO via two reactions: V_o_^+^ + e^−^ → V_o_ or V_o_^2+^ + 2e^−^→ V_o_ [60]. The volatile memory behavior induced by optical stimulation is exclusively attributed to the dynamics of the IGZO channel layer. Since wavelengths of approximately 275 nm or shorter are essential to induce a photoresponse in wide‐bandgap materials with energy gaps greater than 4.5 eV, such as HZO and Si_3_N_4_, the 405 nm laser, which corresponds to a photon energy of 3.06 eV, selectively ionizes only the oxygen vacancies within the IGZO. Indeed, it has been reported that HfO2 and Si_3_N_4_ exhibit an extinction coefficient that saturates at 0 in the 405 nm regime, essentially precluding light absorption and allowing total transmission of the incident light, which confirms that the underlying dielectric layers act as completely optically transparent media [61, 62]. Figure 4c shows the trends of peak current and decay time as a function of intensity using a 3D plot. The optical pulses used for the measurement varied in intensity from 8.15 to 37.34 mW cm^−2^, all with a fixed 4000 ms pulse width. The highest peak of 362 pA was obtained at 37.34 mW cm^−2^, suggesting that more photocurrent was generated by the optical pulse. During the close time, the photoelectrons begin recombination, starting from the nearest shallow traps, and the decay time is determined by the number of electrons returning to deep traps. Figure 4d,e presents the ST decay after the optical peak, confirmed via retention measured for 9 s under a *V*_gs_ of 0 V. The memory index is based on the current value in dark conditions, and the decay rate was calculated based on the difference in photocurrent at 0 s (A_0 s_) and 9 s (A_9 s_). As the optical pulse amplitude gradually increases, the memory index converges exponentially, signifying a transition from strong short‐term characteristics to weaker ST characteristics. The current response to two pulses was evaluated in Figure 4f,g. Each optical pulse used the same 4000 ms open time and 8.15 mW cm^−2^ intensity at a constant *V*_gs_ of 0 V. The complete paired‐pulse responses for close times of 1.0, 2.0, 6.0, 12.0, 22.0, and 40.0 s can be confirmed in Figure S7a–f. The PPF index, calculated for each close time, similarly shows that the exponential decay affects the peak of the next pulse, and it can be approximated by a double‐exponential function model with values *y*_0_ = 0.09, *B*_1_ = 0.18, *t*_1_ = 1.66, *B*_2_ = 0.13, and *t*_2 _= 11.92. In Figure S8, a detailed analysis of the EPSC response under various optical stimulation conditions was conducted with a *V*_gs_ of 0 V. Figure S8a shows the spike‐amplitude‐dependent plasticity (SADP) measured by varying the intensity from 1.8786 to 11.465 mW cm^−2^ for a fixed number of 10 pulses. A linear EPSC gain was obtained from the peak of the 10th pulse relative to the first, showing enhanced synaptic conductance modulation. Figure S8b shows the spike‐number‐dependent plasticity (SNDP) measured by adjusting the pulse count from 3 to 32 at an intensity of 8.147 mW cm^−2^. The SNDP ratio, determined from the peak of the n_th_ pulse, shows that the post‐synapse is strengthened as the count increases. Figure S8c shows the spike‐duration‐dependent plasticity (SDDP) measured under conditions where the open time, which corresponds to the pulse width, was varied from 1000 to 9000 ms. The EPSC gain, obtained from the accumulated and strengthened synapse due to the increased width, increases linearly. Additionally, spike‐rate‐dependent plasticity (SRDP) was measured in Figure S8d under conditions where the close time, which corresponds to the pulse interval, was varied from 1000 to 9000 ms. Similarly, as the interval decreases, the finally summated synapse is reflected in the EPSC gain, showing a negative linear relationship.

**FIGURE 5:**
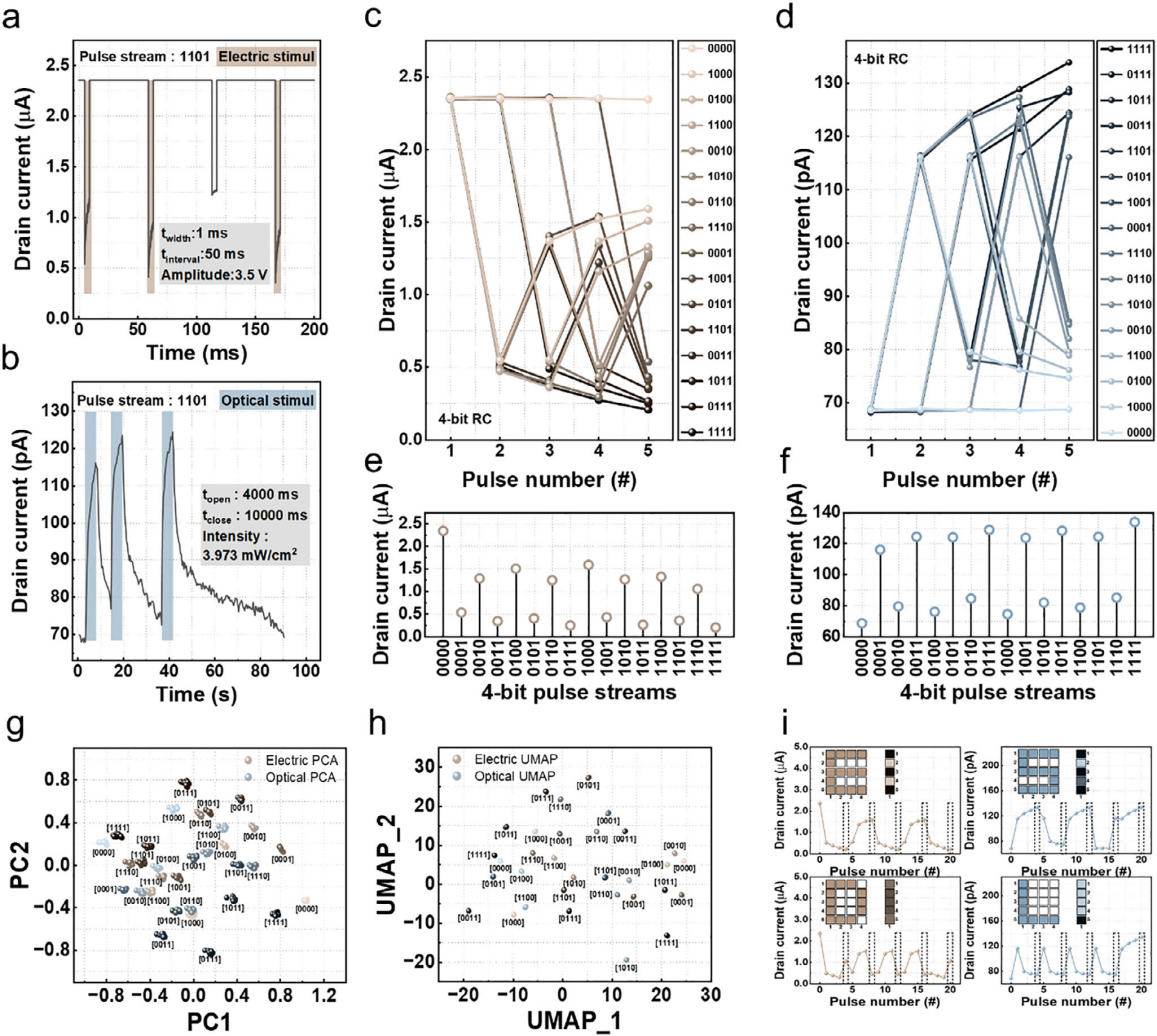
Drain current modulation representing the [1101] state, actuated by (a) electrical stimuli and (b) optical stimuli. (c) 16 distinct states of a 4‐bit reservoir computing system realized via electrical pulses. (d) Corresponding 16 states for the 4‐bit RC system obtained with optical pulses. State separation of the 16 states generated from (e) electrical and (f) optical inputs. Dimensionality reduction of 4D current response vectors using (g) PCA and (h) UMAP for optical and electrical inputs. (i) Mapping of a 2D [ESDL] image using sequential pulses that leverage both short‐term memory characteristics.

In the proposed RC system, the reservoir layer is composed of electric ST and optical ST modes, which serve as non‐linear dynamic nodes to map input time‐varying information into a high‐dimensional feature space. This enables dynamic computation for data processing solely through the intrinsic physical properties of the device, without the need for complex additional circuits. Such hardware‐based high‐dimensional transformation enhances the linear separability of data, thereby drastically simplifying the pattern recognition and training processes and increasing learning efficiency. Consequently, the operation of these two volatile modes enables the implementation of a hardware‐based RC system where a single bit is clearly distinguished as [0] or [1] [63]. Figure 5a shows the [1101] response to electric stimulation, where the pulse for [1] has a 1 ms width and 3.5 V amplitude, and the bit interval is 50 ms. For [0], only a 0.1 V read was performed. The peak characteristic of the [1101] state is a combined form of two PPF index features with 50 and 100 ms intervals, respectively, which can be identified by the peak shift in the raw data. The [1101] state measured for optical stimulation in Figure 5b exhibited an identical trend to the electric [1101]. The [1] pulse used a 4000 ms open time and 3.973 mW cm^−2^ intensity at a *V*_gs_ of 0 V, with a bit close time of 10 000 ms. Figure 5c,d depicts the 4‐bit reservoir states, using the [0] and [1] conditions as a single bit, in the electric and optical environments, respectively. For electric stimulation, a current below the 0.537 µA threshold is distinguished as [1]. For optical stimulation, a threshold of 116 pA exists, and a current above this is clearly distinguished as the [1] state. Individual plots for all reservoir states are presented in Figures S8 and S9. Figure 5e,f illustrates the current values from the final read pulse, corresponding to the 4‐bit streams in sequential order from the [0000] state to the [1111] state, separated by electric and optical inputs. It was confirmed that all expressible 4‐bit states are well separated without overlap. In addition to the fact that the device has 16 clearly separated states, it is essential to verify the fading memory and input‐state stability to prove that it functions as a genuine reservoir rather than a simple multi‐state dynamic system. To confirm this, an additional experiment was conducted where the identical stimulus pattern corresponding to the 4‐bit condition was consecutively applied for 10 iterations (Figure S9a,b). Observing the measured continuous current trajectory of the 10 iterations, the conductance of the device does not accumulate infinitely or saturate despite repeated stimulation. Instead, it perfectly recovers a constant initial current at each cycle, exhibiting an ideal fading memory dynamic where past inputs are appropriately relaxed. Simultaneously, to quantify the cycle‐to‐cycle variation of the peak states extracted from the trajectory, we analyzed the coefficient of variation by applying the formula *CV*  = σ/μ  × 100, where *σ* and *µ* represent the standard deviation and the mean, respectively. The peak current variation under electrical stimulation was extremely stable at 0.13%, and the peak current variation under optical stimulation was also extremely low at 1.63%. The consistent, non‐diverging current trajectory and the extremely low state variation of 1.63% visually and quantitatively prove that the reservoir state does not drift but always converges to a stable initial state, even under long‐term time‐series stimulation. Consequently, these characteristics strongly support that the proposed device structure is a functional and reliable short‐term memory element for hardware‐based reservoir computing. To evaluate the retention of input history and separability capabilities of the device for distinct input patterns, a 4D state vector was defined, where each dimension corresponds to the bit‐wise current response derived from a 4‐bit stimulus stream. The current response for each input pattern was measured over 10 iterations, and principal component analysis (PCA) was performed to visualize the data distribution within the 4D space (Figure 5g). To identify the principal directions of variance, eigen‐decomposition was performed on the covariance matrix to derive eigenvalues and their corresponding eigenvectors. The data were then projected onto the plane defined by the eigenvectors corresponding to the top two eigenvalues *λ*_1_ and *λ*_2_ with the highest explanatory power. The analysis revealed that the first principal component (PC1) represents the total magnitude of accumulated stimuli, primarily reflecting the strength of conductance potentiation driven by the frequency of pulses corresponding to [1]. The second principal component (PC2) reflects the interaction between information loss due to decay and the influx of new stimuli, serving as an axis that distinguishes the temporal context (timing) of the input patterns. Consequently, the data were observed to be distributed along the PC1 axis based on the count of ′0″s and ′1″s, while patterns with the identical number of pulses were dispersed along the PC2 axis. This suggests that potentiation induced by stimuli and decay over time are the dominant factors of variance in the 4‐bit reservoir states. Furthermore, Uniform Manifold Approximation and Projection (UMAP) was applied to analyze the non‐linear dynamics of the device and the local topological structure of the high‐dimensional data (Figure 5h). To preserve the relationships between data points, UMAP defines the conditional probability *p*_*j*|*i*_ in the high‐dimensional input space and the similarity *Q_ij_* in the low‐dimensional embedding space as follows:

(7) $$p_{j|i}=\exp\left(-\frac{\max(0,d\left(x_{i},x_{j}\right)-\rho_{i}}{\sigma_{i}}\right)$$

(8) $$Q_{ij}=\frac{1}{1+a{\left\|y_{i}-y_{j}\right\|}^{2b}}$$

here, *d*(*x_i_*,*x_j_*) denotes the Euclidean distance in the high‐dimensional space, and ρ_*i*_ is the distance from *x_i_* to its nearest neighbor, ensuring local connectivity of the data. σ_*i*_ is a scaling parameter that calibrates the local density of each point, while a and b are constants that control the compactness of clusters in the low‐dimensional space. Through this mathematical modeling, a high‐dimensional fuzzy simplicial set was constructed, and optimization was performed by minimizing the Cross‐Entropy between the two distributions P and Q, thereby preserving the structural relationships of the data. The UMAP visualization confirmed that the 16 distinct 4‐bit input patterns formed clear, non‐overlapping clusters within the 2D space. This indicates that the non‐linear rise and decay characteristics of the device effectively map distinct spatiotemporal input patterns onto unique states within the state space. Moreover, it was observed that states induced by electric and optical stimuli were situated within distinct cluster regions in the UMAP space and exhibited mutual separability, even for identical 4‐bit input patterns. This implies that the device can distinguish not only the spatiotemporal pattern information of input pulses but also the modality of the stimuli, encoding them into separate unique states. Therefore, whether the two stimulation modes are operated independently or utilized simultaneously, an expanded state space can be secured without signal interference. This suggests that the proposed device is suitable for implementing optoelectronic multimodal reservoir computing systems. Figure 5i depicts the process of mapping an alphabet, represented by a 5 × 4 grid, sequentially row by row as 4‐bit inputs. The bits, having two states [0] and [1], are mapped to uncolored and colored, respectively. As a typical example, the letter [S] can be grouped into 1 × 4 matrix block units of [1111], [1000], [1111], [0001], [1111]. If a 4‐bit block is stored in LTM, the conductance change depends only on the number of [1] states. Therefore, the [1000] state is indistinguishable from the [0001] state, which has the same count of [1] s. Consequently, it is impossible to distinguish the letter [E]. In a STM device, however, the conductance change varies not only on the pulse number axis but also in the time domain. The characteristic of state decay, or loss over time, makes it possible to distinguish between [1000], which is stimulation on the first bit followed by decay, and [0001], which is decay followed by stimulation on the last bit. Consequently, unlike LTM, which would require 20 bits or a 4 × 5 grid to store one letter, all alphabets can be represented using only 5 STM block inputs. The alphabet, represented as 1 × 4 matrices and compressed by leveraging the two short‐term mechanisms in the time domain, suggests the potential for the FeTFT to process large‐scale images with more bits and shows its usability as a physical reservoir.

### 2.4 A Fully FeTFT‐Based RC System with Triple‐Mode Operation

A FeTFT based on a Si_3_N_4_/HZO/IGZO tri‐layer is implemented by electrical and optical pulses to operate in three modes, electric LT, electric ST, and optical ST, and is used for the realization of a fully hardware‐based RC system by using the two ST modes as the reservoir layer and the LT mode as the readout layer. The optical short‐term (ST) mode is not redundant with the electrical ST mode; instead, it introduces a distinct and slower temporal dynamic that expands the reservoir state space beyond what is achievable with a single electrical mechanism, thereby enhancing pattern separability in the hybrid reservoir.

**FIGURE 6:**
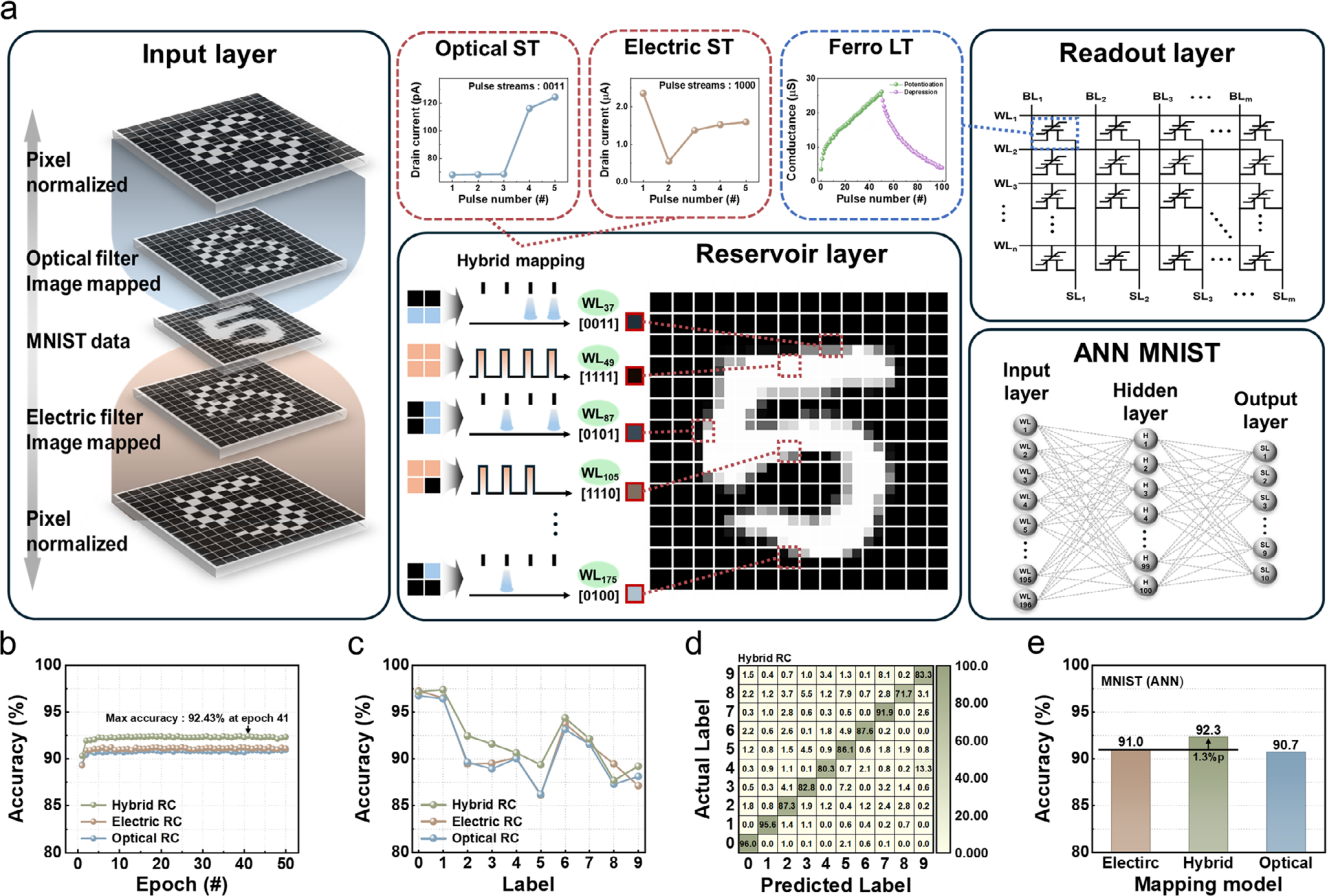
(a) Flowchart of the developed fully triple‐mode FeTFT‐based RC system. The volatile reservoir synapses are controlled by photoelectrons and charge trapping, while the nonvolatile readout synapses are adjusted by ferroelectric dipole switching. (b) MNIST data recognition accuracy per epoch for the three models, using the 2 × 2 matrix and incremental PD. (c) Recognition accuracy for each label, classified using the weights trained by the three models. (d) Confusion matrix of the hybrid RC model. (e) Final MNIST data recognition accuracy comparison for the three mapping models.

To verify the performance of this fully hardware‐based RC, a handwritten digit recognition task was performed using the MNIST database. Figure 6a presents this RC schematic. Using light to create pulses requires additional optical equipment. When transmitting signals to the device using optical equipment, one faces error problems due to noise, such as obstruction. Such input errors can also occur in an electrical pulse environment due to metal line yield issues. To ensure robustness against loss in information transmission, a hybrid mapping model was applied. The MNIST data used for classification consists of a 28 × 28 matrix totaling 784 pixels, with each pixel having a brightness value from 0 to 255. This data was processed by applying an optical filter and an electric filter, followed by mapping using blocks composed of a 2 × 2 matrix. A block composed of a 1 × 4 matrix carries only linear information and causes horizontal distortion in the original input data, leading to a significant decrease in accuracy for digits with similar vertical features, such as [4] or [9]. The 2 × 2 matrix, however, includes forward compression and spatial information, reducing data distortion and improving the clarity of distinctions. Subsequently, to simplify the data size, a pixel normalization process is performed, setting the brightness value to [0] if it is between 0 and 127, and to [1] if it is between 128 and 255. The differences caused by each dataset's filter for randomly selected digits from 0 to 9 are shown in Figure S12a–c. In the reservoir layer, each cell within a block is read sequentially. If the brightness is [0], only a read is performed. If it is [1], an optical or electric pulse is applied according to the filter to create the pulse scheme. The completed 4‐bit pulse enters through the gate line for electrical stimulation or directly into the IGZO via optical equipment for optical stimulation. Finally, based on the channel conductance, the dataset is compressed into a 14 × 14 dataset consisting of 0 and 1 s. The final dataset generated in the reservoir layer is fed as input to the readout layer. In the training phase, the dataset injected via a feedforward method and the weights are used to calculate a weighted sum, which enters the activation function of the hidden layer to finally output a predicted value. A difference exists between the predicted value and the actual value, which is represented by the loss function. To reduce the error, the gradient of the loss function must be reduced, and the gradient can be calculated using the backpropagation method. A weight update occurs to minimize the gradient. If the gradient was negative, the state was raised to increase conductance, if positive, the weight state was lowered via depression. Since the trained weights must be used in actual classification as well as in subsequent training, the electric LT mode was used to adjust these weights.

Figure 6b depicts the training results from 60 000 datasets over 50 epochs in an ANN with a 196 × 100 × 10 architecture simulation. The hybrid model achieved the highest accuracy of 92.43% at epoch 41, followed by the electric and optical models, in that order. The state distinction becomes clearer as the interval between the [0000] and [1111] states becomes more uniform, meaning more linear. The accuracy difference between the electric and optical models is attributed to this linearity of the 4‐bit reservoir states, the electric model exhibited a more linear state distribution than the optical model, resulting in superior state distinction. This linearity is also considered crucial in weight updates, and the accuracy difference between identical and incremental PD is depicted in Figure S13a−c. Weight linearity not only improves learning speed but also adds to the adjustable weight range. Consequently, when using identical PD, the accuracy decreased by 1.69%p during the training phase, and the incremental method showed superior accuracy for all labels. Figure 6c illustrates the classification task performed by label on 10 000 datasets using the trained weights. The hybrid model showed a significant difference in accuracy for all labels except [8]. Confusion matrix of the overall recognition result of the hybrid model, which used the 2 × 2 block and incremental PD, is shown in Figure 6d. Figure S14a−f presents the accuracy difference between applying the 1 × 4 matrix and the 2 × 2 matrix for the three models. Fundamentally, the 2 × 2 matrix not only mitigates distortion and includes spatial information but also increases the possibility of selectable states. A block with a 1 × 4 matrix mostly uses continuous states like [0011] or [0111], besides the most common [0000] and [1111]. In contrast, the 2 × 2 matrix utilizes rarely used states like [0101] and [1001], enhancing the complexity of the input data. This enhancement in complexity is maximized in the hybrid model. This enhancement in complexity is directly reflected in the diversity of the reservoir states. While the 2 × 2 block structure inherently increases spatial diversity, the hybrid model further amplifies this by generating heterogeneous reservoir states through the integration of distinct time constants. Specifically, the electric mode is characterized by fast relaxation times of 0.02 and 0.18 s, whereas the optical mode exhibits significantly slower dynamics with relaxation times of 1.6 and 11.92 s. These distinct temporal properties allow the reservoir layer to map identical input patterns to different state values depending on the mode, thereby enriching the reservoir state space. To quantitatively verify this expansion of dimensionality, we analyzed the average ER for 100 randomly selected MNIST samples, calculated as:

(9) $$\mathrm{ER}\;=\;\exp\left(-\sum_{k=1}^{N}p_{k}\log p_{k}\right)$$

(10) $$p_{k}=\frac{\sigma_{k}}{\sum_{i=1}^{N}\sigma_{i}}$$

where p_k_ represents the normalized energy distribution derived from the singular values σ_k_ of the reservoir state matrix. The simulation results demonstrated that the single‐mode models exhibited an average ER of approximately 6.1, whereas the hybrid model achieved a significantly higher average ER of 11.29. This nearly two‐fold increase confirms that the stochastic integration of fast electrical and slow optical dynamics maximizes the information capacity by diversifying the reservoir states. Before being modulated by the filter, it can have 30 states per block. Figure 6e summarizes the final recognition accuracy for each mapping model. The hybrid model achieved a peak accuracy of 92.3%, representing a 1.6%p improvement over the optical model [64, 65]. This 1.6%p increase is comparable in magnitude to the 1.69%p accuracy gain obtained by optimizing the weight update linearity. Such a performance enhancement is attributed to the proposed tri‐layer architecture, which maximizes dynamical diversity by integrating heterogeneous time constants from fast charge trapping and slow optical ionization. These results suggest that coupling distinct physical mechanisms within a single device provides a meaningful approach to elevating the performance of hardware‐based RC systems. Table 1 compares the device characteristics of this study with recently researched 3 terminal TFT based RC systems. This study is unique in its demonstration of a TM FeTFT device, which implements three modes, electric LT, electric ST, and optical ST, within a single device, originating from electrical/optical stimuli and three distinct layers without additional processes. This system utilizes the electric ST and optical ST modes as a hybrid mapping model in the reservoir layer, and employs the electric LT mode as the hardware readout layer instead of using simulation, thereby implementing a fully hardware‐based RC. In terms of performance, this system achieved a peak fully hardware‐based RC accuracy of 92.3%, which is higher than readout results using other ST‐TFTs with a single ST device in ANN methods, excluding CNN methods. To evaluate the minimum power consumption (*P*_min_) for each model during the read operation, *P*_min_ was obtained by the product of the minimum reservoir state current and the constant read voltage. Based on this evaluation, the electric model recorded a *P*_min_ of 20.06 nW, while the hybrid model integrating both mechanisms exhibited an average power consumption of approximately 10.04 nW. Notably, when operating under the optical model, the device recorded an ultra‐low minimum power consumption of 11.6 pW, reducing it by approximately two times compared to the previous lowest power consumption of 22 pW [71]. Additionally, the device secured balanced linearity factors of *L*_P_ = 1.282 and *L*_D_ = 0.683. These results demonstrate the superiority of the proposed TM FeTFT‐based RC system compared to previous studies.

**TABLE 1:** Benchmark of our fully TM FeTFT‐based RC system and comparisons with other TFT‐based published studies.

Device stack (gate/gate insulator/channel)	RC		*MW*	Linearity		Power consumption (optimal performance)	Full hardware‐based RC system? (yes or no)	Accuracy (4‐bit MNIST)	Refs.
	Reservoir layer	Readout layer		*L*_P_	*L*_D_				
W/HfO_2_ HfO_2‐x_/IGZO FeTFT	Electric ST mode	Simulation	2.09 V	—	—	≈6.25 nW	No	94.29% (CNN)	[66]
Cr/Hybrid AlO_x_/Al_2_O_3_/MoS_2_ TFT	Electric ST mode	Simulation	2.4 V	—	—	≈3.5 nW	No	93.7%	[67]
Si/SiO_2_/Al_2_O_3_/SnO TFT	Electric ST mode	Simulation	2.4 V	—	—	≈25 nW	No	94.1%	[68]
Au/CuInP_2_S_6_/h‐BN/α‐In_2_Se_3_ FeTFT	Electric ST mode	Simulation	4.72 V	—	—	≈0.8 nW	No	89.46%	[69]
Si/HZO/IGZO FeTFT	Optical ST mode	Electric LT mode	2.8 V	2.37	0.57	≈ 0.5 nW	Yes	90.5%	[70]
TiN/HZO/IGZO FeTFT	Optical ST mode	Electric LT mode	1.5 V	1.32	0.64	≈22 pW	Yes	88.01%	[71]
Mo/ZrO_2_/IGZO/ZrO_2_/HZO/Mo FeTFT	Electric ST mode	Electric LT mode	2.2 V	1.02	0.49	≈180 nW	Yes	90.23%	[72]
TiN/Si_3_N_4_/HZO/IGZO FeTFT	Electric ST mode /optical ST mode	Electric LT mode	5.46 V	1.28	0.68	≈11.6 pW	Yes	92.3%	This work

## 3 Conclusion

In this study, we successfully implemented three independent memory modes in a single FeTFT device based on a Si_3_N_4_/HZO/IGZO tri‐layer stack, and integrated them to propose and verify a fully hardware‐based RC system. The electric LT mode, based on the ferroelectric polarization of HZO, showed a wide memory window of 4.93 V, an endurance of 10^5^ cycles, and multi‐state retention characteristics exceeding 5000 s. Notably, by introducing the incremental pulse scheme and effectively decoupling the transient ST interference via sufficient read delays, the linearity was drastically improved, enabling the linear weight adjustment essential for the RC readout layer. The electric ST mode, based on charge trapping/detrapping in the Si_3_N_4_ trap layer, demonstrated its operating mechanism through low‐voltage CW hysteresis and PPF characteristics. The optical ST mode, based on the optical ionization/recombination mechanism in the IGZO channel, also exhibited short‐term retention, PPF, and various synaptic plasticities such as SADP, SNDP, SDDP, and SRDP. In the MNIST handwritten digit recognition task, the hybrid system, using these two electric/optical ST modes as the reservoir and the LT mode as the readout, achieved a high recognition accuracy of 92.43%. The ability to compositely leverage heterogeneous physical mechanisms within a single device confirms the FeTFT's viability as a fundamental building block for high performance neuromorphic systems.

## 4 Experimental Section

Figure S15 shows the detailed fabrication process of the FeCAP device. An n^+^ Si substrate with a resistivity above 0.005 Ω cm was cleaned with a solution mixed with a 4:1 ratio of H_2_SO_4_:H_2_O_2_ to effectively remove organic contaminants. The native oxide was removed using a diluted HF solution (HF:H_2_O = 1:100). Subsequently, a 100 nm thick TiN film was deposited by DC sputtering to serve as the gate electrode. Following this, 2 nm of Si_3_N_4_ was formed at 350°C using an OXFORD plasma enhanced chemical deposition. A 5% SiH_4_/N_2_ mixed gas and NH_3_ reactant gas were used. A 7 nm HZO ferroelectric layer was formed at 290°C using a CN1 atomic layer deposition. Tetrakis hafnium (TEMAH) and tetrakis zirconium (TEMAZ) were used as precursors, respectively, and O_3_ was used as the oxidant. The final HZO composition was controlled to a 1:1 molar ratio of HfO_2_ and ZrO_2_. Afterward, a 100 nm tungsten layer was sputtered via physical vapor deposition (PVD) as a capping layer. To introduce tensile stress, RTA (KVT‐3006T) was performed at 500°C for 30 s under an N_2_ atmosphere, after which the capping layer was removed by wet etching. Ta was then deposited via DC sputtering and subsequently patterned to form the top electrode. Figure S16 shows the fabrication process of the FeTFT device. An additional step of depositing a 100 nm thick TiN film and then patterning it to form the gate was included. After the wet etching of the W capping layer, a 20 nm IGZO layer was deposited via radio frequency sputtering and then patterned to form the channel. The IGZO layer is composed of In, Ga, and Zn in a 1:1:1 molar ratio. Ta was deposited via DC sputtering and patterned to form the source/drain electrodes. The thin film thickness and elemental composition were confirmed using TEM and EDS. The electrical and optical characteristics of the fabricated FeTFT were measured using Keithley 4200‐SCS and 4225‐ PMU modules.

## Author Contributions

Hyeonho Lee was responsible for conceptualization, data curation, formal analysis, and writing – original draft. Tae‐Hyeon Kim validated the results and participated in writing – review and editing. Seungjun Lee, Yonghee Jeong, Gwangmin An, Minsu Ko, and Junsu Yu contributed to data curation, resources, software, and writing – review and editing. Heung Soo Kim validated the results and responsible for funding acquisition. Yang Chai participated in writing – review and editing. Sungjun Kim provided methodology and resources and participated in writing – review and editing. All authors discussed the results and contributed to the final version of the manuscript. Sungjun Kim was responsible for funding acquisition.

## Conflicts of Interest

The authors declare no conflicts of interest.

## Supporting information




**Supporting File**: advs75471‐sup‐0001‐SuppMat.docx.

## Data Availability

The data that support the findings of this study are available from the corresponding author upon reasonable request.
