# Supplementary material for: Triple‐Mode Ferroelectric Thin‐Film Transistor for Hybrid Electrical–Optical Reservoir Computing
Source: Adv Sci (Weinh). 2026 May 11;13(42):e75471. doi: 10.1002/advs.75471 (PMC13335677; doi:10.1002/advs.75471)
Supplement: Supplementary file 1 — Supporting File: advs75471‐sup‐0001‐SuppMat.docx. [file ADVS-13-e75471-s001.docx]

Supporting Information


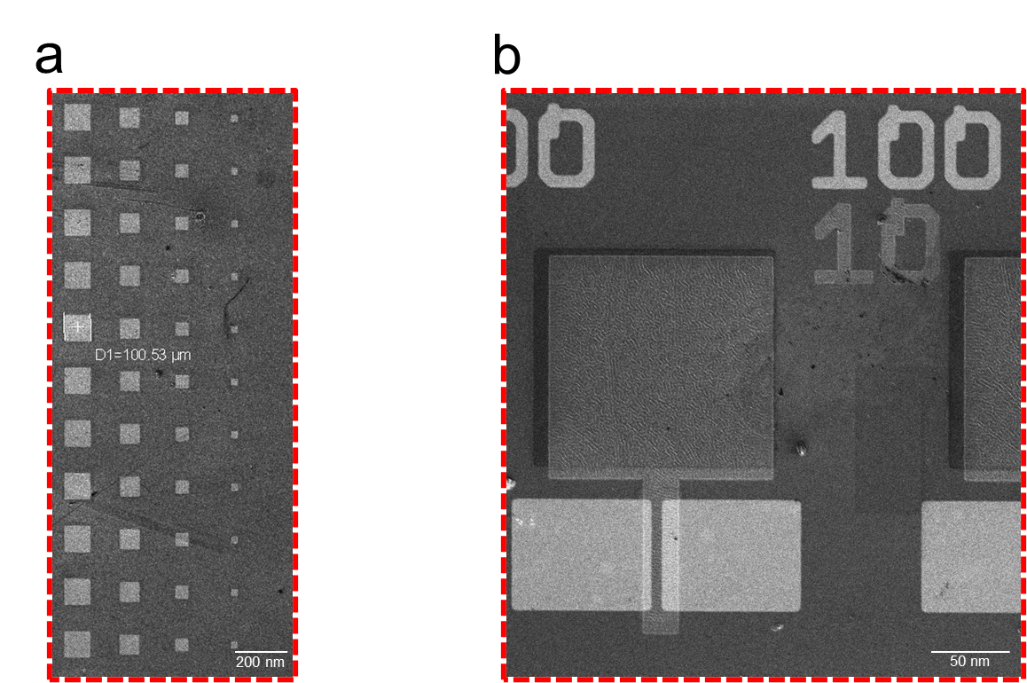


**Figure S1.** CD-SEM images of the fabricated (a) FeCAP and (b) FeTFT devices.


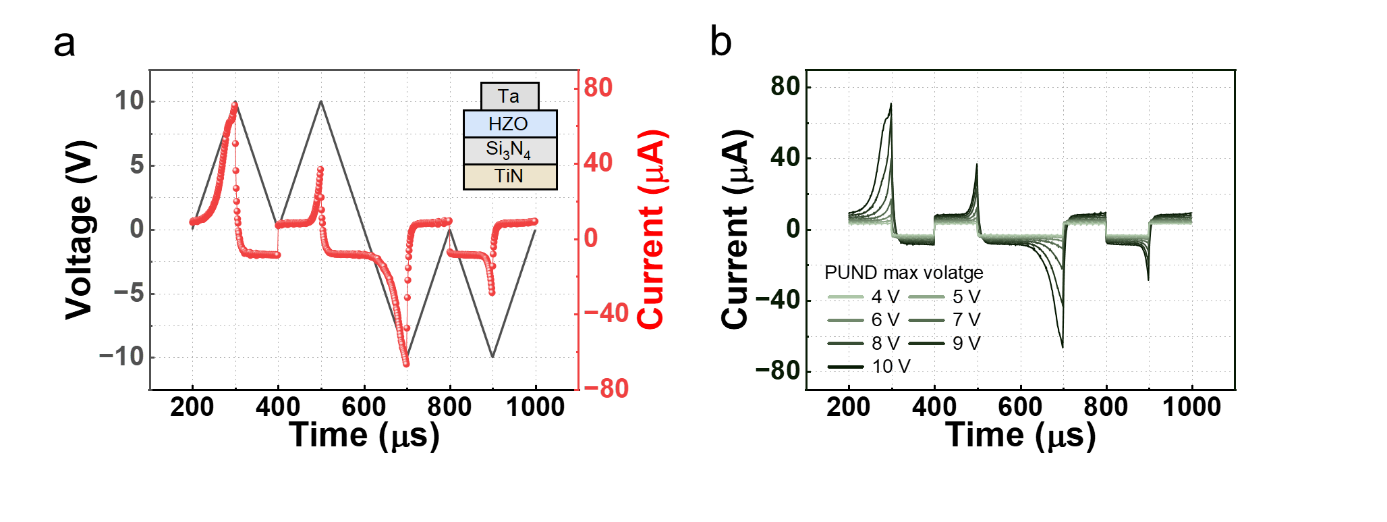


**Figure S2.** a) The 10 V PUND pulse scheme and the overlapped current response of the MIFM capacitor. b) Dependence of the switching current on the PUND maximum voltage, ranging from 4 V to 10 V in 1 V increments.


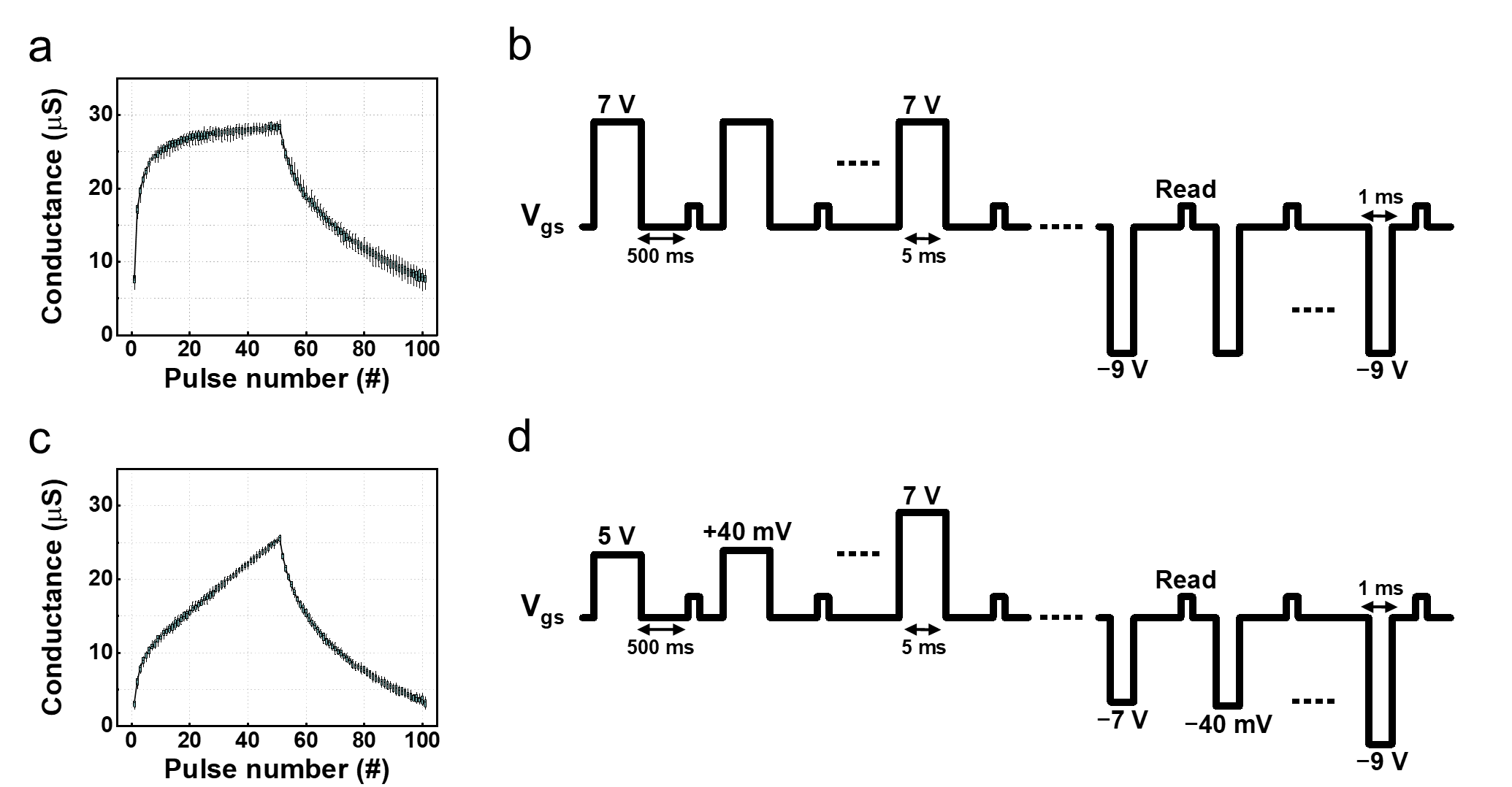

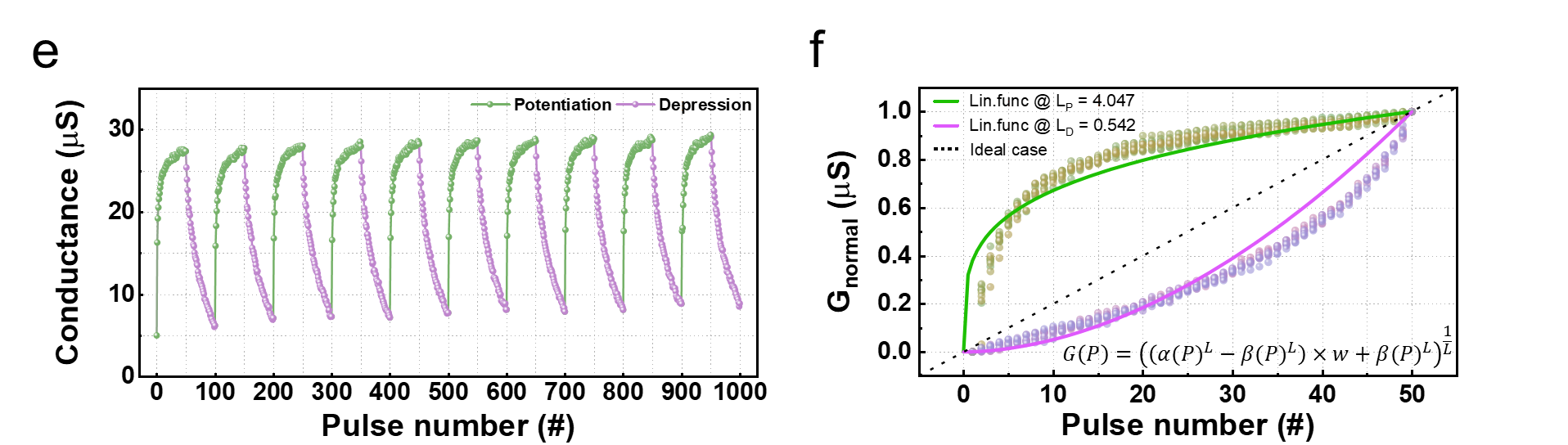


**Figure S3.** a) Stability at each pulse number for the 10 cycles identical PD. b) The overall identical pulse scheme applied to the TiN gate. c) Cycle-to-cycle stability for the 10 cycles incremental PD. d) Schematic of the overall incremental pulse scheme applied to TiN gate. e) Electrical LTP and LTD measured using 10 cycles identical pulse scheme. f) Normalized and overlapped 10 cycles PD, and the linearity function approximated from the conductance data for the identical pulse scheme.


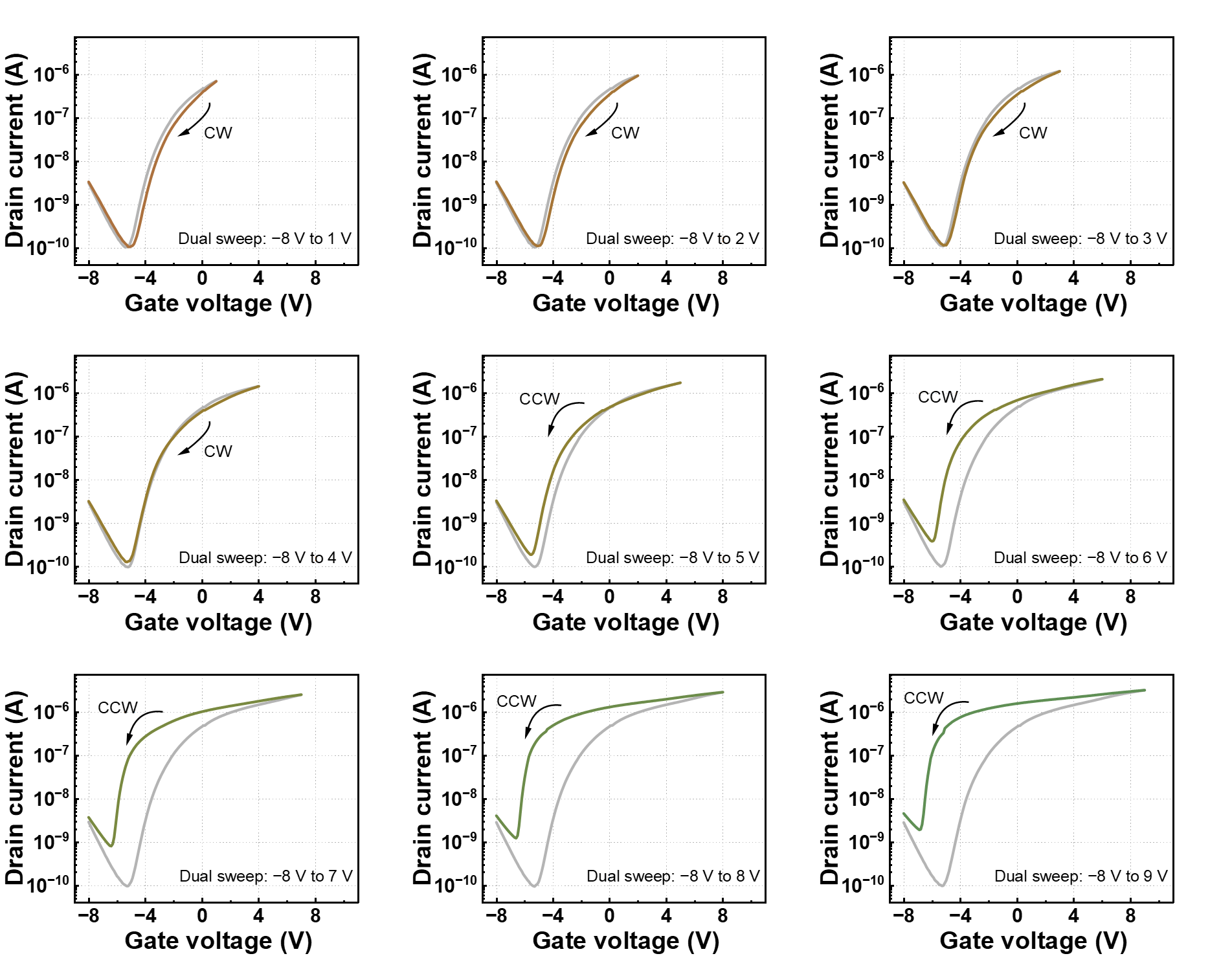


**Figure S4.** Plot of the dual-sweep I_ds_-V_gs_ transfer curves for the FeTFT, with V_gs_ swept from −8 V to varying V_max_ ranging from 1 V to 9 V in 1 V increments.


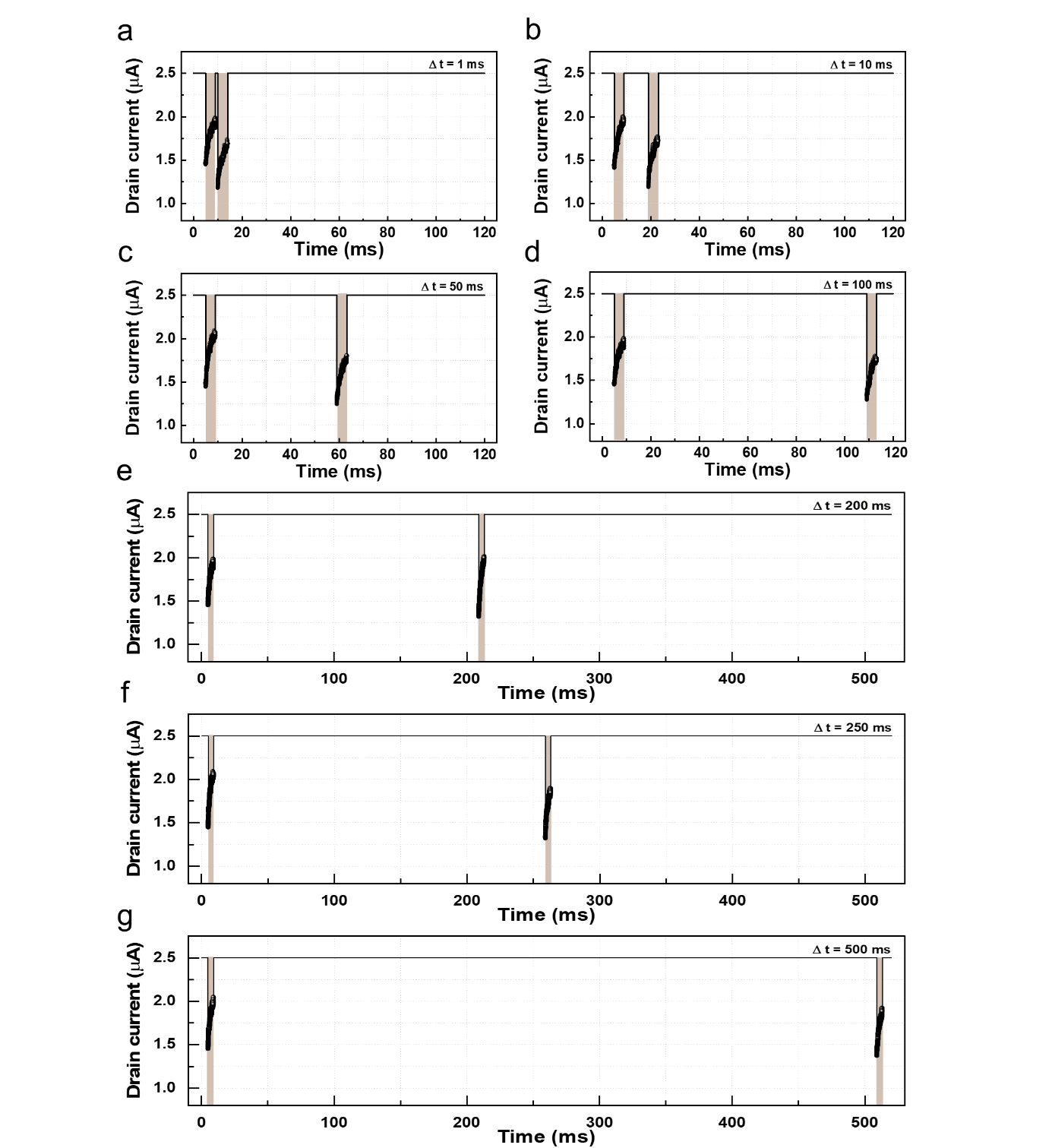


**Figure S5.** Results of electric PPF measurements at various pulse intervals a) 1 ms, b) 10 ms, c) 50 ms, d) 100 ms, e) 200 ms, f) 250 ms, and g) 500 ms.


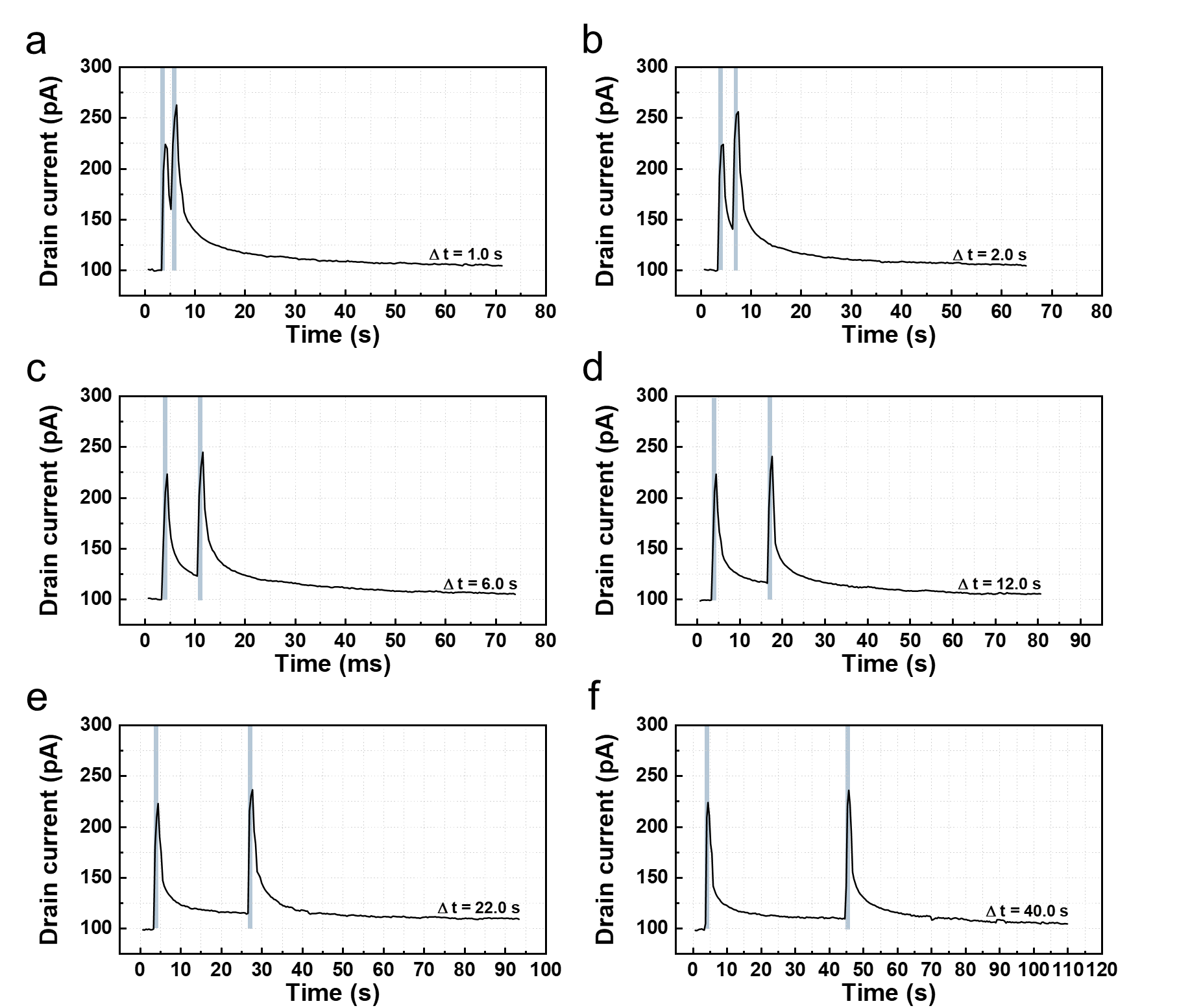


**Figure S6.** Results of electric PPF measurements at various pulse intervals a) 1.0 s, b) 2.0 s, c) 6.0 s, d) 12.0 s, e) 22.0 s, and f) 40.0 s.


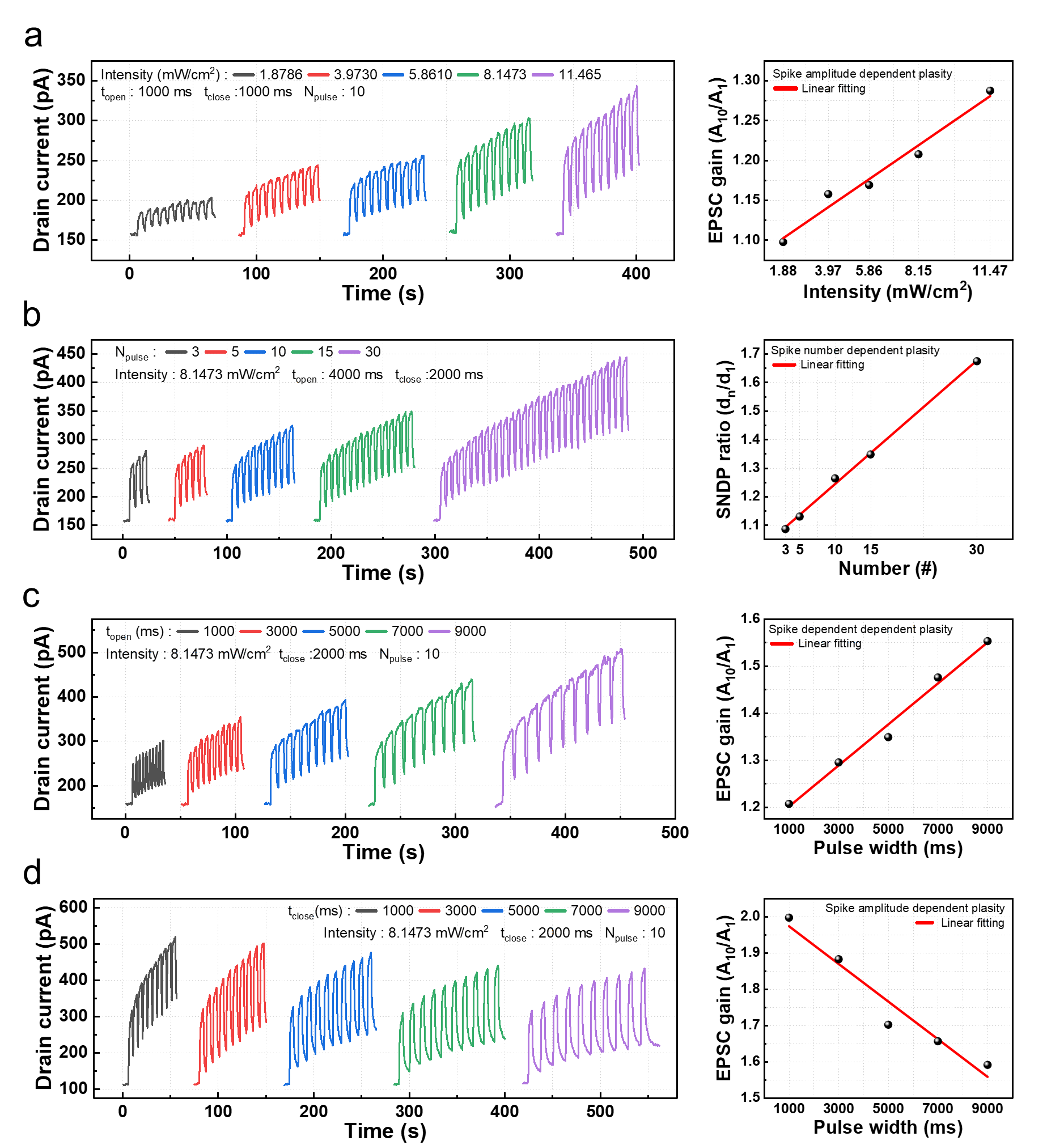


**Figure S7.** a) Weight variation depending on the intensity of optical pulses, along with EPSC gain as a function of optical pulse intensity. b) Short-term synaptic plasticity of the device, demonstrated by synaptic weight modulation by optical pulse number, and the SNDP ratio according to the number of optical pulses. c) Changes in weight with respect to the duration of optical pulses and the corresponding EPSC gain plotted against optical pulse width. d) Weight modulation as a function of the interval of optical pulses and EPSC gain versus optical pulse intervals.


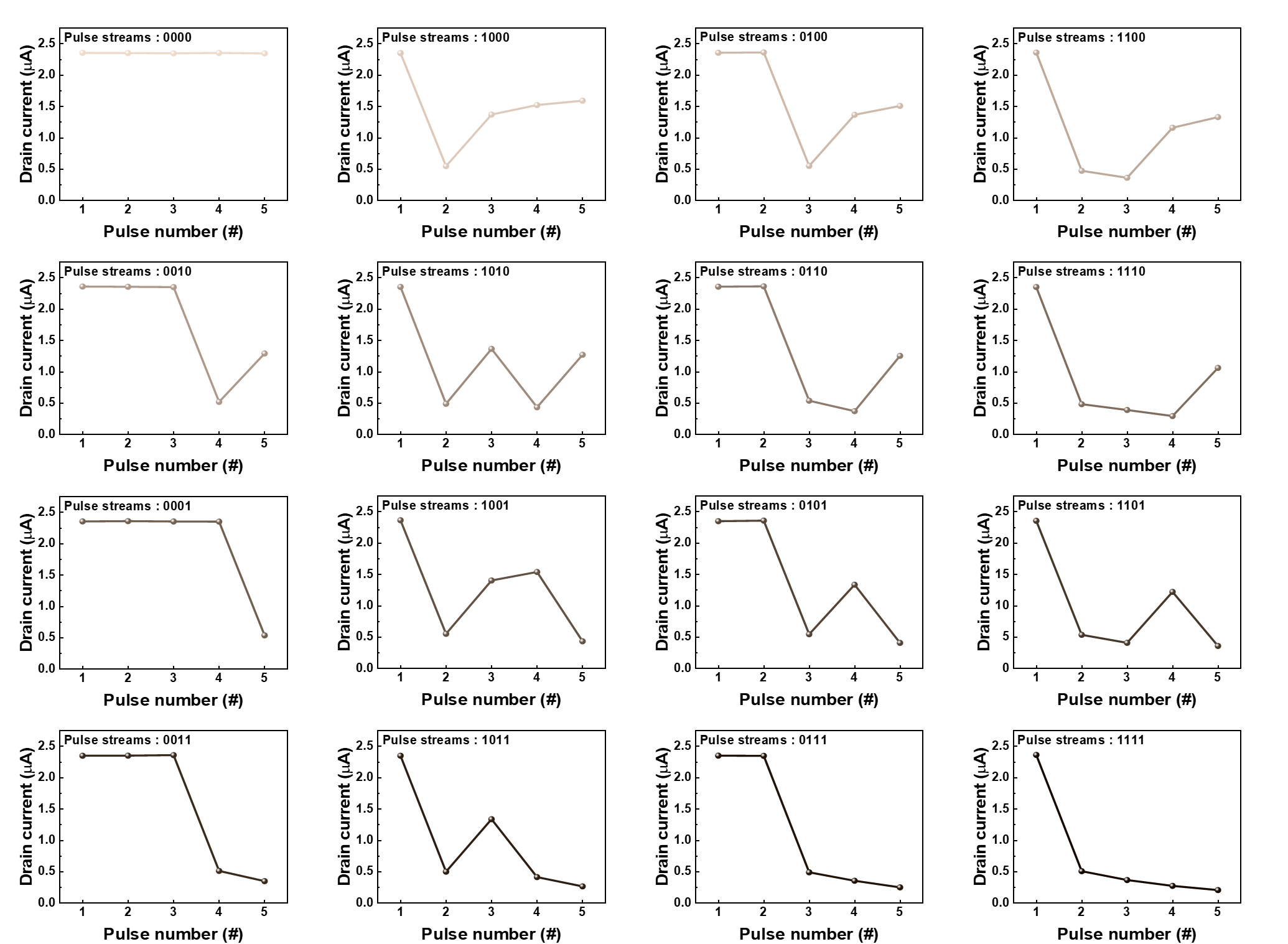


**Figure S8.** The 16 electric states of the 4-bit RC system, ranging from [0000] state to [1111] state.


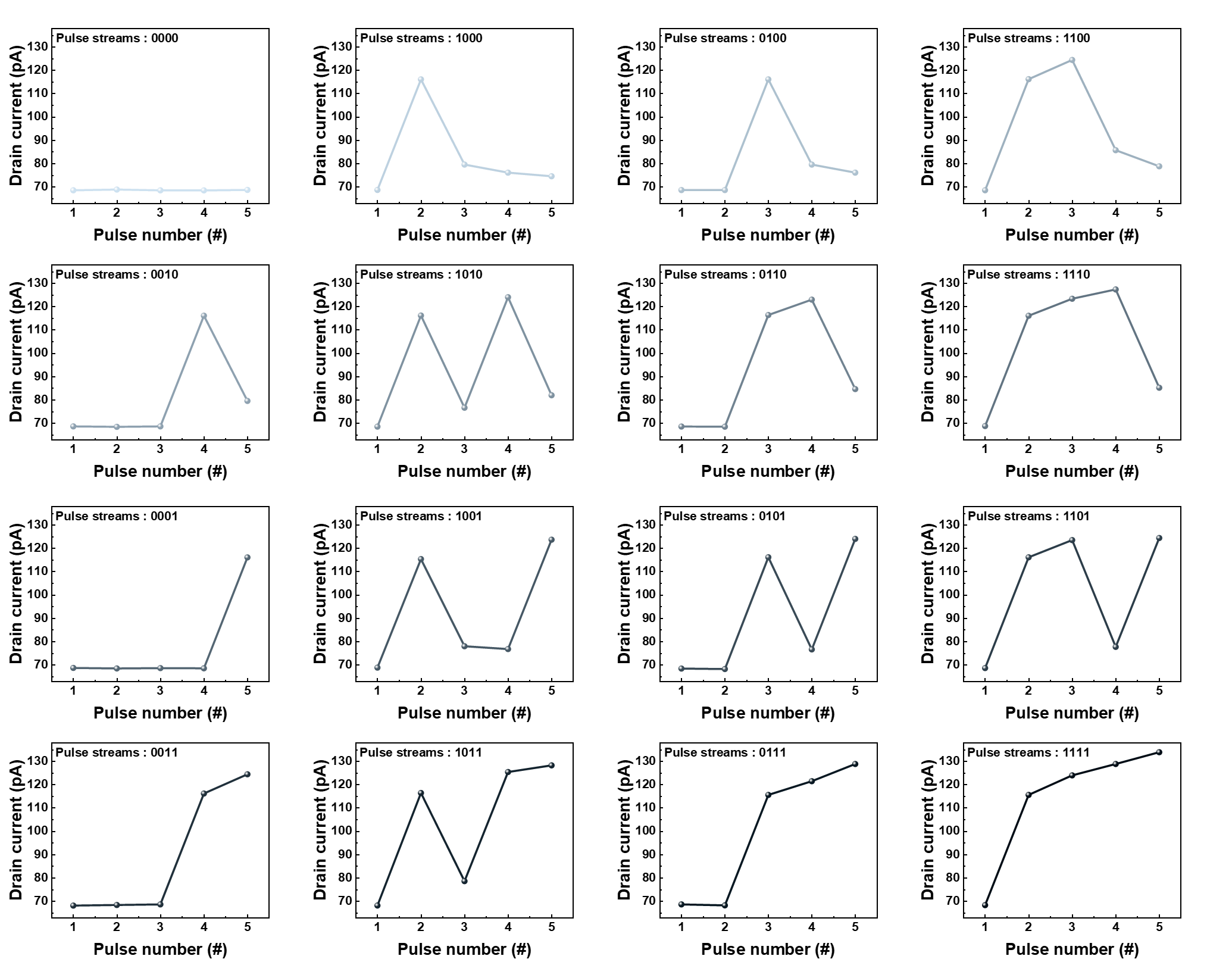


**Figure S9.** The 16 optical states of the 4-bit RC system, ranging from [0000] state to [1111] state.


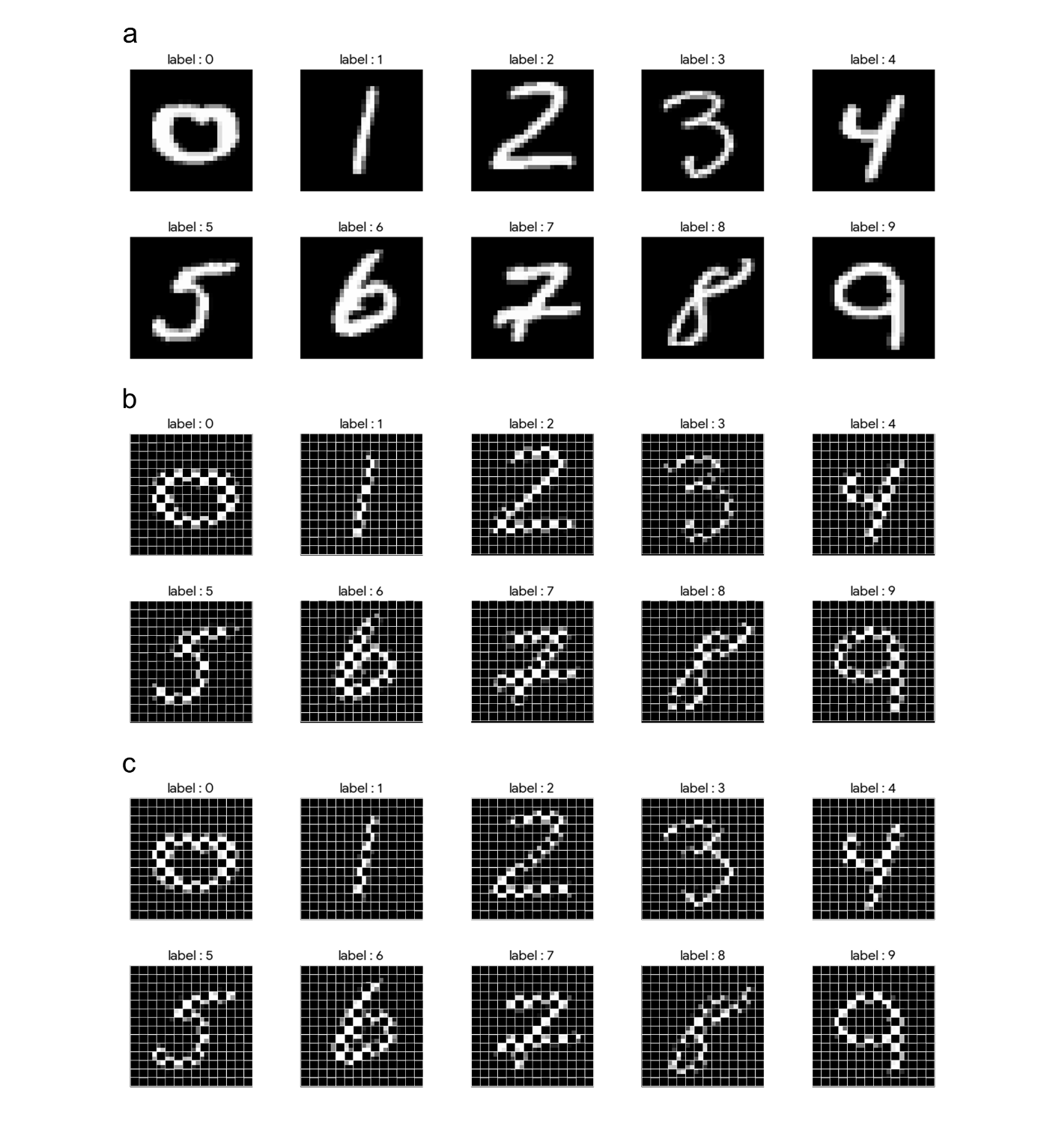


**Figure S10.** a) Original randomly selected MNIST samples (labels 0-9). b) MNIST samples processed with an optical filter (labels 0-9). c) MNIST samples processed with an electric filter (labels 0-9).


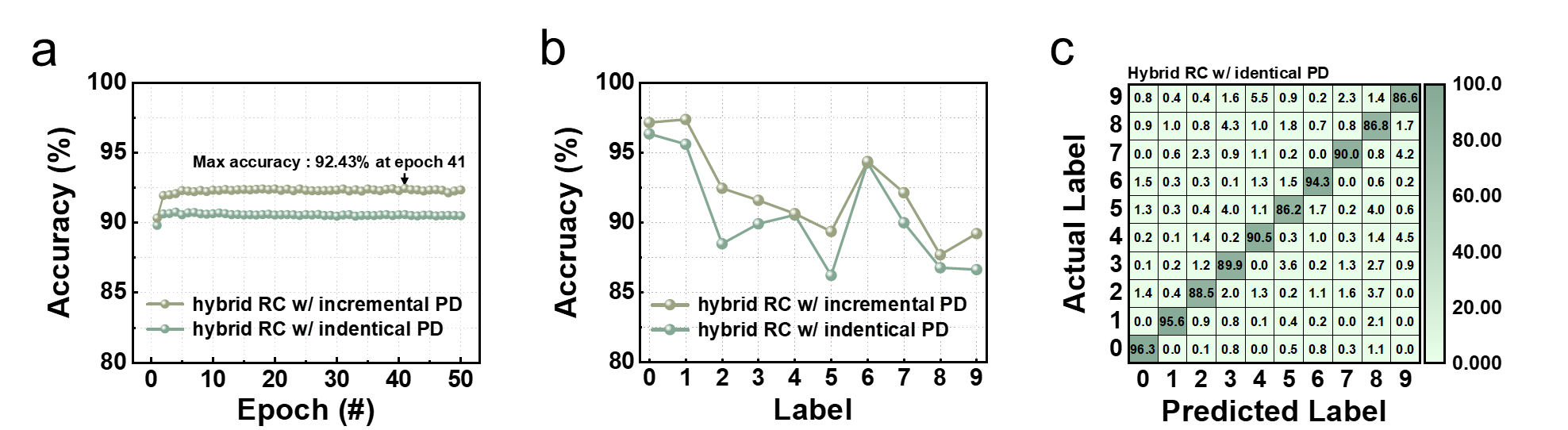


**Figure S11.** a) MNIST data recognition accuracy per epoch for the identical and incremental pulse schemes (using the 2×2 matrix). b) Recognition accuracy for each label, classified using the weights trained by the two different pulse schemes. c) Confusion matrix of the hybrid RC model trained using the identical pulse scheme.


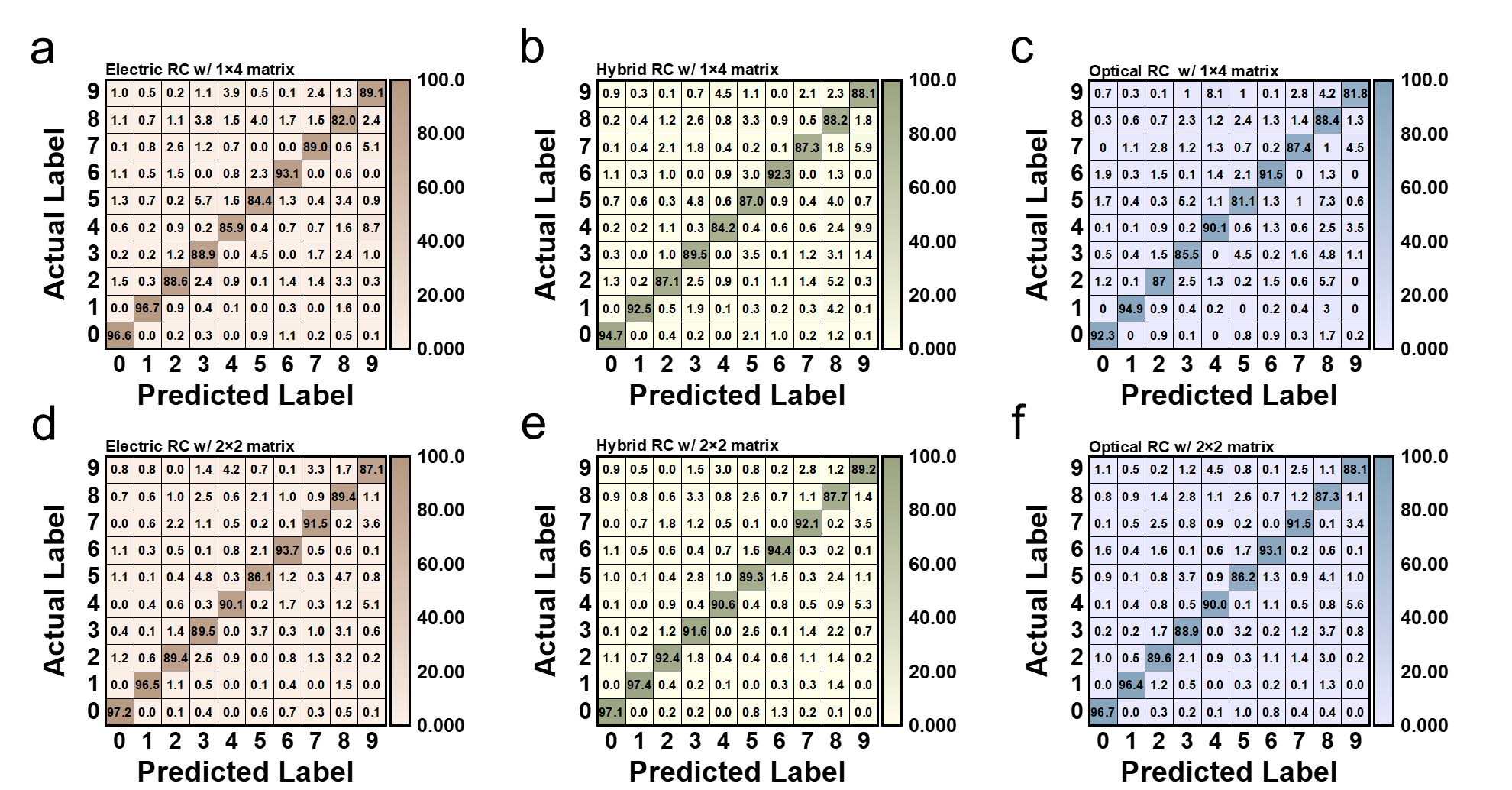


**Figure S12.** Confusion matrices of the a) electric, b) hybrid, and c) optical RC models trained using the incremental pulse scheme with a 1×4 matrix input. Confusion matrices of the d) electric, e) hybrid, and f) optical RC models trained using the incremental pulse scheme with a 2×2 matrix input.


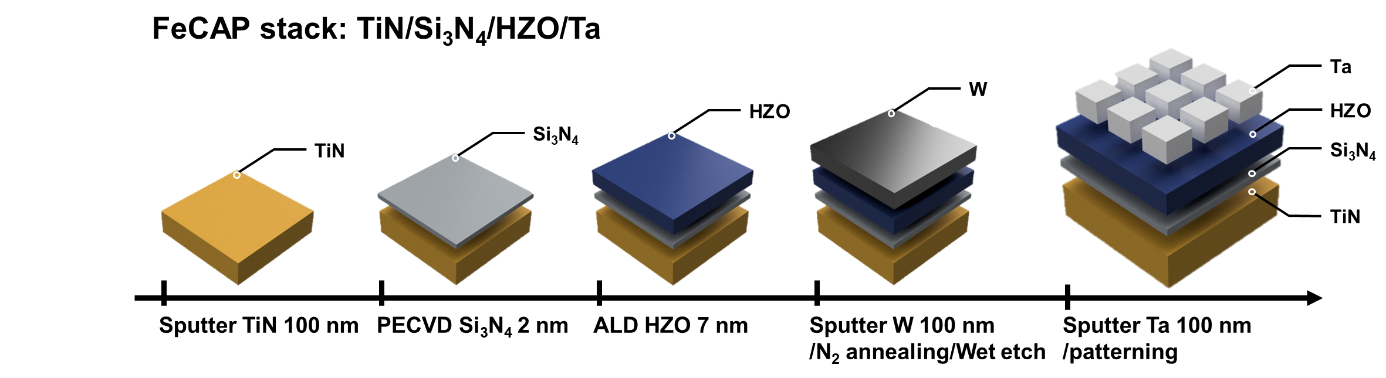


**Figure S13.** Detailed fabrication process flow of the TiN/Si_3_N_4_/HZO/Ta FeCAP.


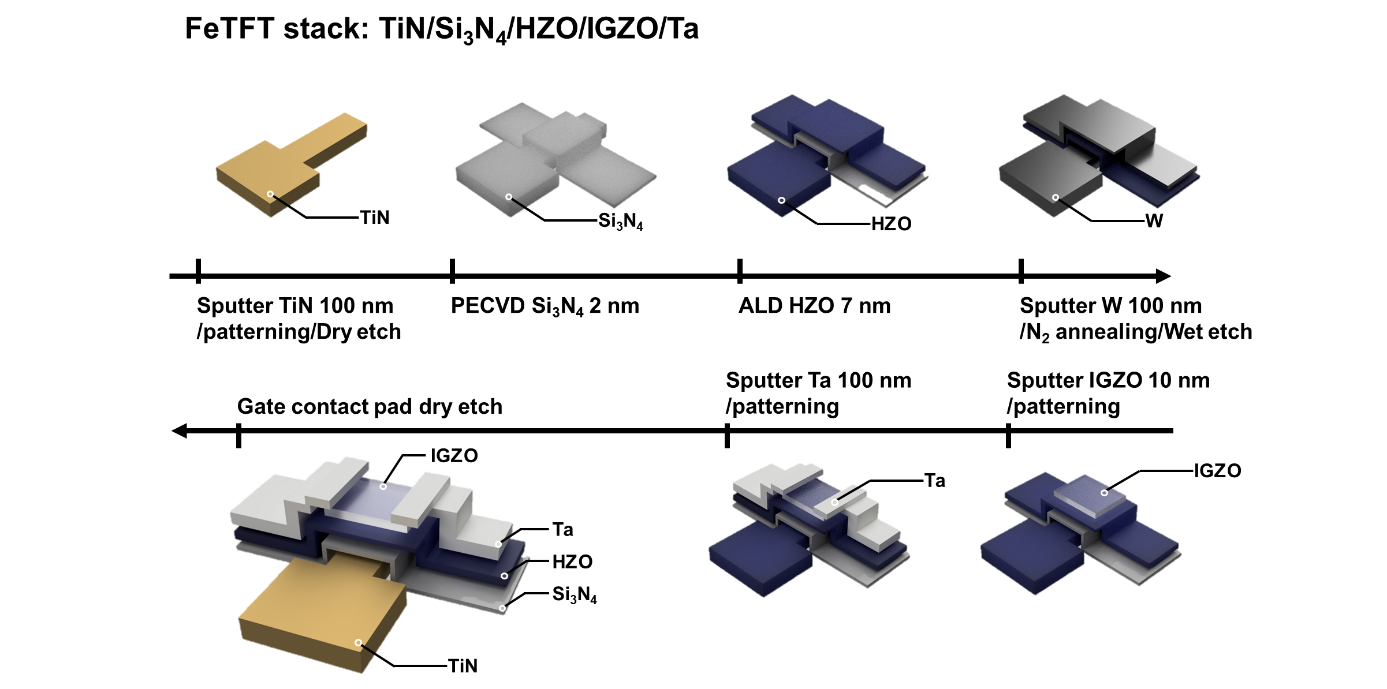


**Figure S14.** Detailed fabrication process flow of the TiN/Si_3_N_4_/HZO/IGZO/Ta FeTFT.
